# TIME Is Ticking for Cervical Cancer

**DOI:** 10.3390/biology12070941

**Published:** 2023-06-30

**Authors:** Vijay Kumar, Caitlin Bauer, John H. Stewart

**Affiliations:** 1 Department of Interdisciplinary Oncology, Stanley S. Scott Cancer Center, School of Medicine, Louisiana State University Health Science Center (LSUHSC), 1700 Tulane Avenue, New Orleans, LA 70012, USA; 2 Louisiana Children’s Medical Center Cancer Center, Stanley S. Scott Cancer Center, School of Medicine, Louisiana State University Health Science Center (LSUHSC), 1700 Tulane Avenue, New Orleans, LA 70012, USA

**Keywords:** cervix, cervical cancer, HPV, immune cells, inflammation, TIME

## Abstract

**Simple Summary:**

Cervical cancer (CC) is a major health problem in women of childbearing age. For example, CC is the fourth most common cancer in women across the world with an estimated 604,000 new cases in 2020. CC comprised 342,000 deaths worldwide, mainly in women in low- and middle-income countries (LMICs). Frequent CC screening and human papillomavirus (HPV) vaccination programs have significantly decreased the incidence of CC in the developed world. However, the last three years of the coronavirus disease-19 (COVID-19) pandemic have significantly increased CC incidence by disrupting the vaccination program. Understanding the immunological network and environment in the CC tumor microenvironment (TME) called the tumor immune microenvironment (TIME) will help to design immune cell-specific immunotherapeutic approaches for CC patients at different stages. The current article introduces the epidemiology socioeconomic burden of CC, the immune microenvironment in the cervix of healthy women, and its involvement in CC immunopathogenesis. Later sections discuss different immune cells, which comprise CC TIME and its targeting for immunotherapeutic approaches. Therefore, it is critical to understand CC TIME to save our women’s lives from CC throughout the world.

**Abstract:**

Cervical cancer (CC) is a major health problem among reproductive-age females and comprises a leading cause of cancer-related deaths. Human papillomavirus (HPV) is the major risk factor associated with CC incidence. However, lifestyle is also a critical factor in CC pathogenesis. Despite HPV vaccination introduction, the incidence of CC is increasing worldwide. Therefore, it becomes critical to understand the CC tumor immune microenvironment (TIME) to develop immune cell-based vaccination and immunotherapeutic approaches. The current article discusses the immune environment in the normal cervix of adult females and its role in HPV infection. The subsequent sections discuss the alteration of different immune cells comprising CC TIME and their targeting as future therapeutic approaches.

## 1. Introduction

Cervical cancer (CC) is a common cancer affecting women worldwide. For example, 604,000 women across the world suffered and 342,000 women died (mainly in low- and middle-income countries (LMICs)) from CC in 2020 [1]. Although it can be prevented, it still affects an average of 7.7 out of every 100,000 women in the US and is a leading cause of death in young women [1]. In recent years, the incidence of CC in US women between 30 and 34 years old has increased, reaching 11.60/100,000 in 2019 [2]. Unfortunately, the incidence of stage IV CC is higher among Black women as compared to White women [3] and the cost of treating CC is approximately $56,250 for the first year alone [4].

Squamous-cell carcinoma (SCC) accounts for 80% of cases, while cervical adenocarcinoma (CAC) is less common but more severe, making up 15% of all cases [5]. Lifestyle factors can also impact the risk of CC, with an increased number of full-term pregnancies associated with a higher incidence of high-risk precancerous lesions. Additionally, prolonged use of oral contraceptives is associated with high-risk lesions and invasive CC [6]. However, the most significant risk factor for CC is human papillomavirus (HPV), which is associated with 99.7% of all cases [7,8]. Although the introduction of HPV vaccines in 2006 has likely helped reduce the prevalence of CC, it remains a significant cause of cancer deaths [9].

Recent studies have shown that the Cervarix HPV vaccine (GlaxoSmith Kline) protects females from specific HPV-induced CC but may increase their susceptibility to HPV genotypes not targeted by the vaccine [10]. In addition, women who receive the vaccine may still develop grade 2 or 3 cervical intraepithelial neoplasia (CIN) due to nonpreventable HPV types at higher rates than unvaccinated women [11,12,13]. Despite the strong correlation between CC, HPV infection, and the immune system, little is known about the tumor immune microenvironment (TIME) and potential novel CC immunotherapeutics. Therefore, it is critical to understand the local immune environment in the cervix and its role in preventing CC. This review explores the role of various immune cells in CC pathogenesis, its TIME, and the potential for successful immunotherapeutic treatments.

## 2. The Immune Environment of the Healthy Cervix

**Figure 1:**
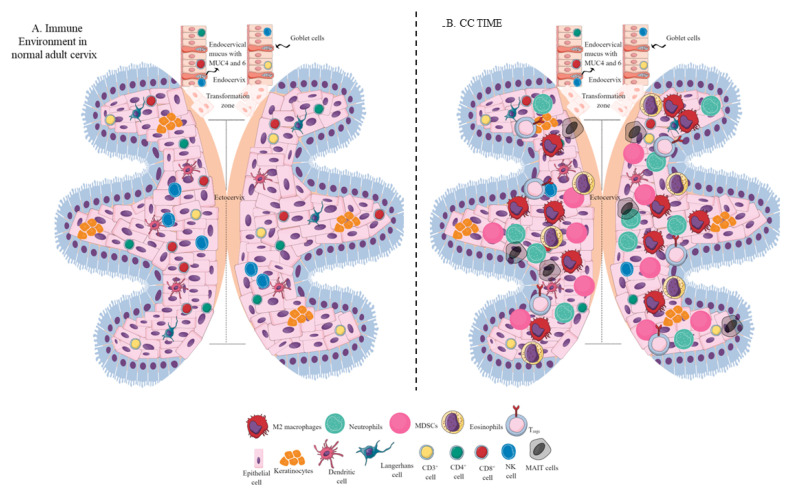
**Schematic representation of the immune-cell population in the normal adult cervix and CC TIME**. (**A**) Immune-cell population in the normal adult human cervix. The cervix is divided into ecto- and endocervix. Endocervix is rich in mucus and glandular goblet cells. Endocervix opens in the uterus. The zone connecting the endo- and ectocervix is called the transformation zone (TZ). Ectocervix is rich in innate immune cell population (Keratinocytes, LC, DC, macrophages, and NK cells) due to its increased chances of exposure to potential pathogens. However, the T-cell population does not vary in the endo- and ectocervix. (**B**). Immune-cell population in the CC TIME. CC TIME supporting tumor growth and metastasis becomes immunosuppressive and TANs, TAMs (M2 macrophages), MDSCs, tDCs, and T_regs_ predominate it. On the other antitumor Th1 and cytotoxic CD8^+^ T and NK cells decrease in number. Details are mentioned in the text.

The cervix is a vital part of the female reproductive tract (FRT) that connects the uterus to the vagina and it is crucial in maintaining pregnancy by protecting against invading microbes [2]. It has two distinct parts: the endocervix, which leads to the uterus and contains glandular cells, and the ectocervix, the outer part of the cervix that opens into the vagina and comprises squamous cells. The transformation zone (TZ), where the endocervix and ectocervix meet, is the most common location for CC to originate from, and its position changes with age and number of pregnancies (Figure 1A). The FRT, similar to the gastrointestinal tract, relies on mucus and mucins for innate immune functions. Therefore, protecting against invading microbes is a crucial function of the cervix, particularly during pregnancy [4,5,6].

The endocervix is protected from pathogens and supports reproductive function through mucus-producing glands known as pseudoglands with goblet cells. On the other hand, the ectocervix, which has low mucus and is frequently exposed to external pathogens, is more vulnerable to microorganisms. This results in a unique immune environment [7,8,9]. Mucin 4 (MUC4), MUC5 (5A and B), and MUC6 are significant components of the endocervical mucus, critical innate immunity components found at mucosal surfaces, including the FRT [10,11,12]. The cervix also expresses various pattern recognition receptors (PRRs), including toll-like receptors (TLRs) such as TLR1 to TLR9 [13,14]. In addition, nucleotide-binding oligomerization domain (NOD) proteins, NOD1 and NOD2, are present in cervical epithelial cells (CECs) and are critical for inflammasome signaling to release proinflammatory IL-1α, IL-18, and IL-33. The cervix with its CECs also has many other humoral innate immune components, including complement proteins, different antimicrobial peptides (AMPs, including defensins), lysozyme, and lactoferrins, along with secretory antibodies (IgA and IgG) of the adaptive immune system. These components complement the mucus-associated innate immunity against invading pathogens, including HPV [15,16,17].

Epithelial cells, particularly CECs, serve as crucial innate immune cells that act as a physical barrier against pathogens in the upper FRT (uterus, fallopian tubes, and ovaries) to prevent ascending infections (as shown in Figure 1A) [15,18,19]. Specialized immune cells in the area also provide innate and adaptive immune responses [15]. CECs play an additional role in promoting the immunological functions of epithelial cervicovaginal Langerhans cells (LCs) and dendritic cells (DC) to fight against invading microbes/pathogens (as shown in Figure 1A). Keratinocytes in the ectocervix also act as innate immune cells, expressing various PRRs such as different TLRs, NOD-like receptors (NLRs), C-type lectin receptors (CLRs), absent in melanoma-2 (AIM-2), and stimulating interferon genes (STING), a downstream adaptor protein for the cyclic guanosine-monophosphate (GMP)-adenosine monophosphate (AMP) synthase (cGAS)/STING signaling pathway (as shown in Figure 1A) [20,21,22,23,24,25].

Cytokines released from activated keratinocytes are known as cytokinocytes. Syndecan-1, a transmembrane heparan sulfate proteoglycan (HSPG) that carries heparan-sulfate (HS) and chondroitin-sulfate glycosaminoglycans on its ectodomain, is also present in ectocervix keratinocytes [26,27]. Syndecan-1 regulates various cellular events, including migration, adhesion, proliferation, and growth, by acting as a coreceptor for different growth factors, cytokines, and chemokines [26]. Interferons (IFNs) secreted by cervical fibroblasts stimulate IFN-stimulated genes (ISGs), indicating cervical immune cell involvement in innate immunity by recognizing and responding to pathogens [28]. Cytotoxic T-lymphocytes (CTLs) in the cervix play a critical protective role and maintain their high levels despite menstrual status [28,29]. However, CD3^+^, CD4^+^, and CD8^+^ cell populations are significantly smaller in the endocervix than in the ectocervix (as shown in Figure 1A). Conversely, NK cell populations do not differ significantly between the endo- and ectocervix (as shown in Figure 1A) [30]. Therefore, even in a healthy cervix, immune cells (CECs, DCs, and T cells) have a dynamic composition (as shown in Figure 1A).

The population of immune cells varies between the cervix and the rest of the FRT [31]. Women who are healthy and do not have any reproductive tract infections or inflammation tend to have many antigen-presenting cells (APCs) and T cells in their cervical TZ. Leukocytes are more common in the upper reproductive tract than the cervix, and macrophages are less common in the cervix than in other tissues, comprising only 10% of all leukocytes in the reproductive tract [28,32,33]. During menstruation and childbirth, the cervix dilates, making the upper reproductive tract particularly vulnerable, and a flexible immune response is necessary [15]. The immune system is programmed to respond by recruiting macrophages to the cervix as it softens during labor [34,35]. Depletion of CD8^+^ T, B, and NK cells following menopause suggests some hormonal regulation on the cervical immune environment [15]. However, not all cervical immune cells are influenced by menstrual status. For example, CD8^+^ T cell activity decreases in the fallopian tubes and endometrium during the secretory phase of the menstrual cycle but remains unchanged in the cervix [36]. It is essential to understand the cervical immune landscape to comprehend cervical carcinogenesis.

## 3. CC Immunopathogenesis

CC is mainly caused by high-risk strains of HPV, such as HPV16 and HPV18. This correlation is more significant in women older than 35, as younger women tend to have shorter HPV infections [37]. The type of HPV strain also affects the development of cancerous or precancerous lesions [38]. HPV16 is the most common strain, followed by HPV18 [38,39]. Women with HPV16 infection are at a higher risk of developing CC and more severe lesions than women with HPV18 [40]. HPV16 is especially prevalent in Italian women [41] and Chinese patients with SCC [42], while HPV18 is more common in Iranian women [43]. Nevertheless, HPV infection dramatically increases women’s risk of CC or precancerous lesions.

**Figure 2:**
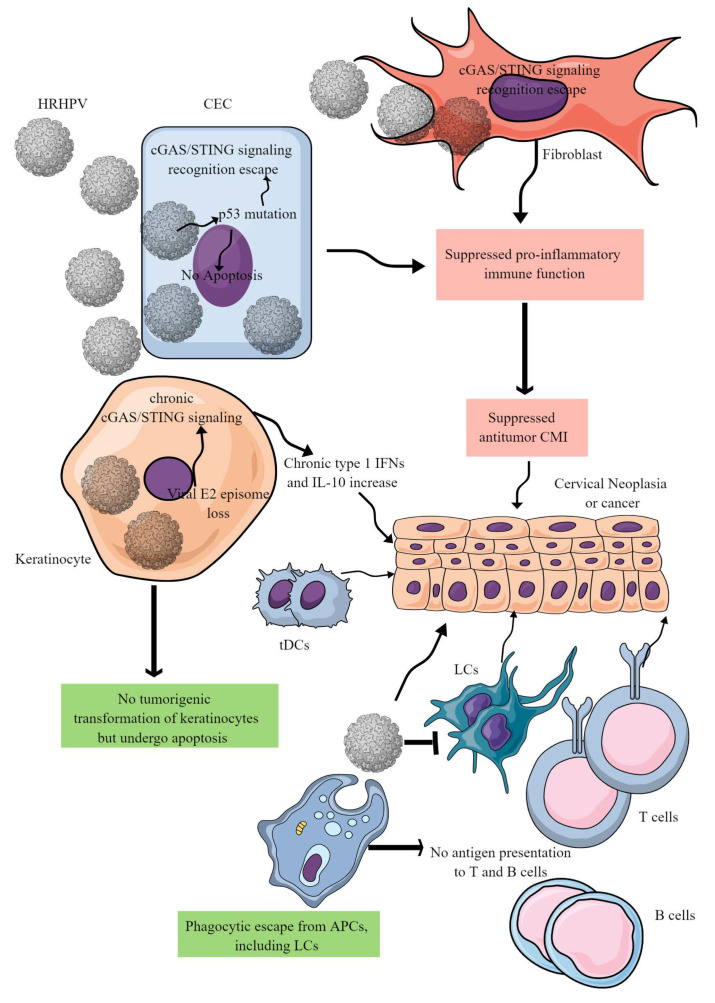
**HR-HPV-mediated CC immunopathogenesis.** HR-HPVs infect basal epithelial cells, keratinocytes, and fibroblasts. HR-HPV infection transforms epithelial cells into neoplastic cells and later into cancer. The other keratinocytes escape from tumorigenic transformation and undergo apoptosis to support the immunosuppressive environment for tumor growth. In epithelial cells, HR-HPV infection prevents their apoptosis by inducing p53 mutation that also inhibits the cGAS/STING-mediated antitumor immune response. In addition, other proinflammatory innate immune functions of epithelial cells are also blocked, creating a chronic tumor-supportive immunosuppressive niche. Notably, in keratinocytes, only cGAS/STING-mediated NF-κB-mediated release of proinflammatory cytokines is inhibited leaving the type 1 IFN generation intact. This leads to the chronic type 1 IFN generation, which supports tumor growth. Furthermore, HR-HPV escapes phagocytosis by antigen-presenting cells (APCs, macrophages, LCs, and DCs) and antigen presentation to T and B cells. Metabolites released in the TIME by cancer cells further suppress cDC1s and NK cells. For example, DCs repolarize to tDCs in the presence of tryptophan and its metabolism by IDO. Details are discussed in the text.

The lifecycle of high-risk HPV is discussed elsewhere [44,45,46], but this section focuses on its immunopathogenesis and immune escape mechanisms. HPV infection suppresses the immune response and thereby increases the risk of CC through escaping cancer immunosurveillance and cancer immunoediting. Authors have discussed cancer immunosurveillance failure and cancer immunoediting elsewhere in detail [47]. For instance, HPV implies different strategies to escape immune surveillance for initiating and progressing the CC. For example, after infecting epithelial cells, HPV DNA integrates into gene-dense chromosomal regions responsible for transcriptional activities [48]. HPV can remain latent for years without clinical manifestation [49] and, during this time, it suppresses the host’s immune response. The epithelial-cell infection of HPV hijacks its innate immune-cell function and suppresses the proinflammatory immune response that is required to generate cell-mediated immunity (CMI) to clear the HPV infection (Figure 2) [50,51]. This characteristic of the HPV replicative cycle has been exquisitely shaped by its coevolution with the host [52] and enables HPV to avoid immune recognition and clearance.

HPV primarily avoids immune recognition during the initial infection phase by escaping the cytosolic cGAS/STING signaling-mediated recognition in epithelial cells and keratinocytes due to its unique vesicular trafficking pathway (Figure 2) [53]. Additionally, cervical keratinocytes with HPV16 are refractory to tumorigenic transformation by oncogenic H-Ras (Harvey rat sarcoma virus, a cellular GTPase that signals through mitogen-activated protein kinases (MAPK), phosphatidylinositol 3 kinase (PI3K), and Ral-GEF pathways expression (Figure 2) [54]. Along with the genetic integration of HPV DNA with the host cell DNA, the loss of the E2 episome (circular viral DNA that remains unintegrated and competent for viral transcription and replication during latency loss) is critical for cervical carcinogenesis [55,56].

During HPV infection of cervical keratinocytes, the loss of the E2 episome chronically stimulates antiviral genes. This stimulation is induced by the cGAS/STING signaling-dependent proliferation of type 1 IFNs and NF-κB-responsive genes, which promotes a chronic proinflammatory environment that supports tumor growth [55,57]. The HPV 16 and 18 E7 oncoprotein inhibits this signaling and the release of type 1 IFNs in CECs, keratinocytes, and fibroblasts (Figure 2) [57,58], while blocking cGAS/STING-dependent NF-κB-mediated immune responses. However, E7 does not affect IRF3 activation responsible for type 1 IFN generation in CECs (Figure 2) [59]. Chronic cGAS/STING-dependent type 1 IFN release can have a protumorigenic effect, as evidenced by the association of the rs311678 polymorphism in the cGAS gene with increased genetic susceptibility to HPV-induced precancerous cervical lesions [60]. E6 increases the synthesis and release of various cytokines in differentiating cervical keratinocytes, including IL-8, regulated upon activation, normal T cells expressed and secreted (RANTES or CCL5), macrophage inflammatory protein-1α (MIP-1α or CCL3), and interferon-γ-induced protein-10 (IP-10 or CXCL10) [57]. Keratinocytes also produce IL-10 to create a tumor-supportive chronic inflammatory environment (Figure 2) [61]. E6 and E7 further enhance this environment by inhibiting the release of transforming growth factor beta (TGF-β) from keratinocytes [62]. The HPV16 E7 oncoprotein can cause cervical keratinocytes to become more sensitive to apoptosis and release type 1 IFNs, leading to a chronic tumor-supportive inflammatory environment [63]. However, HPV16 E7 in CECs can prevent apoptosis and support uncontrolled growth and cancer development (Figure 2) [64]. HR-HPVs can evade phagocytosis by APCs and the antigen presentation to T and B cells [52], decreasing the chances of systemic immunity development. HPV can also suppress apoptosis and initiate squamous intraepithelial lesion development (Figure 2) [65]. In addition, p53 loss or mutation can suppress STING activation, which is critical to induce apoptosis of the infected cell and the release of type 1 IFNs for antiviral and antitumor immunity [66].

Patients with CC overexpress the regulator of G protein signaling 1 (RGS1) oncogene [67], which can inhibit proinflammatory immune-cell infiltration, reduce Th1 and cytotoxic CD8^+^ T-cell survival, and promote T-cell exhaustion, leading to an immunosuppressive environment [67,68,69,70]. RGS1 overexpression in CC correlates with increased HPV^+^ E6 in AC and cervical SCC, contributing to rapid cancer progression [67]. However, RGS1 knockdown can inhibit tumor growth, migration, and proliferation by promoting cancer cell apoptosis [67]. Therefore, targeting RGS1 has excellent potential for adjunct cancer immunotherapy for patients with CC. In addition, differential gene expression is associated with metabolic and hypoxic pathways and immune-cell activation and infiltration, altering CC risk and progression [71].

The E6 and E7 oncoproteins play a significant role in cancer progression caused by HPV. When absent, CC cells undergo apoptosis [72]. In addition, HPV16-E7 stimulates CCL5 and CCL6 chemokines, attracting mast cells to create an immunosuppressive microenvironment that supports tumor growth [73]. These viral oncoproteins contribute to six key characteristics of a cancerous environment, including resisting cell death, inducing angiogenesis, and triggering metastasis [74]. In addition, E6 and E7 inhibition induces aging among HPV^+^ CC cells and reactivates the tumor-suppressor gene p53 and other antiproliferative proteins [75]. Thus, E6 and E7 inhibitors are attractive therapeutic targets to explore.

LCs are potent myeloid innate immune cells in the skin and other epithelial environments, including stratified epithelia of the corneal, buccal, gingival, and genital mucosae [76,77,78]. HPV 16 virus-like particles (VLPs) called HPV16-L1 and L2 have been shown to avoid recognition by LCs and suppress their innate immune function to avoid the antiviral immune response (Figure 2) [79]. HPV 16 interacts with the annexin A2 hetero-tetramer (A2t) to infect the basal epithelial cells (BECs) and LCs. Blocking A2t with HPV16 L2 prevents HPV-mediated LC maturation suppression, as demonstrated by increased secretion of Th1-associated cytokines and surface expression of MHC class II on LC [79]. LCs in the cervix also serve as critical innate immune cells against HPV infection. They can present HPV antigens to induce an adaptive T-cell immune response when treated with different immunomodulators including poly I:C, 3M-002(TLR8 agonist) and resiquimod (TLR7/8 agonist) [80,81]. In addition, chimeric HPV VLPs (HPV cVLPs) can potentially activate LCs and DCs.

The activation of LCs and DCs is indicated by the upregulation of surface activation markers and an increase in the secretion of IL-12p70. This activation is critical for antiviral/antitumor NK and Th1 cells and increases IFN-γ [82,83,84]. The HPV-16 L1-VLPs recognition by DC surface antigens (human leukocyte antigen class 1, or HLA class I) and many cytokines/chemokines, particularly TNF-α, IL-6, and RANTES (regulated on activation normal T cell expressed and secreted), is mediated by DC-specific intercellular adhesion molecule-grabbing nonintegrin (DC-SIGN) [85]. HPV cVLPs have great potential for designing CC vaccines, but LC and DC stimulation with HPV without costimulation is insufficient to activate residential T cells [86]. Adjuvants added to HPV cVLPs can induce antiviral and antitumor T cell-mediated immunity against HR-HPVs, making it a novel immunotherapeutic approach for CC.

**Figure 3:**
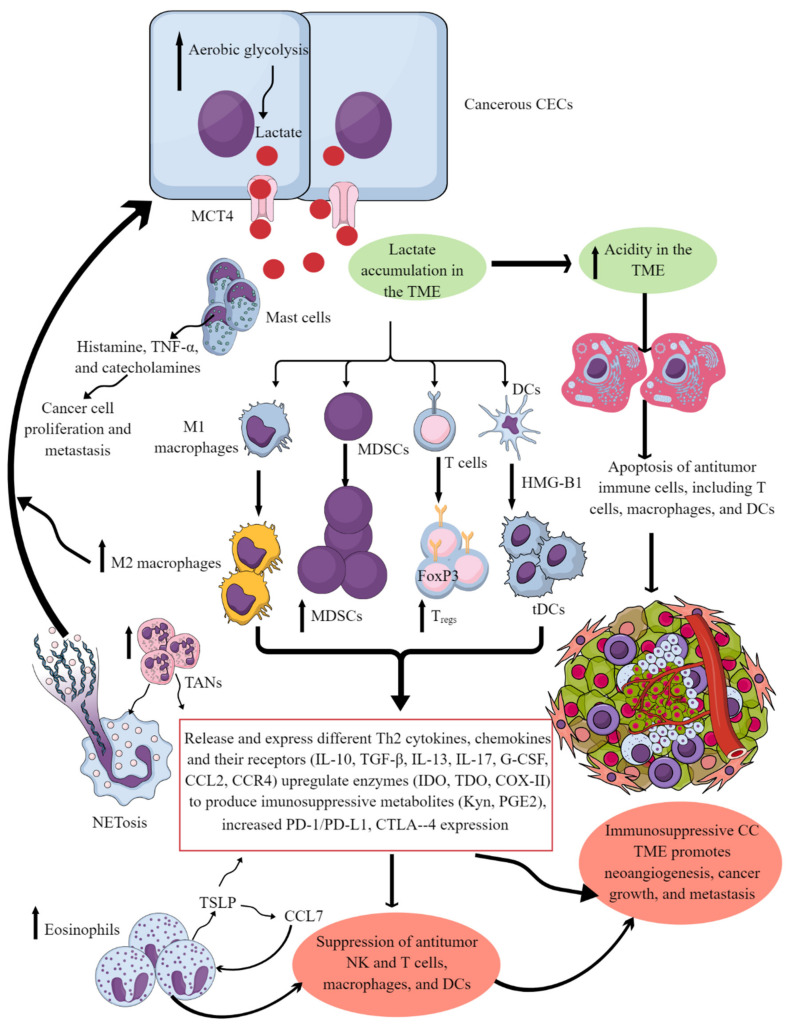
**Cervical cancer and Immune cells crosstalk to create and support the immunosuppressive CC TIME.** Cancerous CECs with increased aerobic glycolysis (due to increased energy demand to maintain their rapid growth and proliferation) overproduce lactate. This lactate is released into the TME or TIME to create an acidic microenvironment. The acidic TME induces apoptotic cell death of different antitumor immune cells and reprograms their immunometabolism to polarize them to tumor-supportive immunosuppressive immune cells (M1 to M2 macrophages, increase in MDSCs, TANs, T_regs_, and tDCs). These immunosuppressive immune cells synthesize, express, and release different tumor-promoting immunosuppressive molecules for their growth and proliferation along with supporting the cancer growth, proliferation, and metastasis. These immune cells also release angiogenic factors to support neoangiogenesis for cancer survival and metastasis. Eosinophils and mast cells release several factors (TSLP, TNF-α, histamine, and catecholamines) to support immunosuppressive CC TIME and cancer-cell proliferation and metastasis. Details are mentioned in the text.

In CC, cellular metabolism plays a critical role in carcinogenic immunometabolic reprogramming. Patients with CIN and ICC due to HR-HPV infection show altered glucose transporter 1 (GLUT1), lactate dehydrogenase A (LDHA), and monocarboxylate transporter type 4 (MCT4, lactate transporter) [87]. LDHA is overexpressed in HPV16^+^ CIN patients, and GLUT1 expression is higher in CIN-stage I patients than in a control group, which further increases in CIN-II/III patients’ cancer cells. This increased expression indicates increased glucose utilization by glycolysis in CC cells, creating a glucose-deprived niche for immune cells. The overexpression of LDHA and MCT4 is more prevalent in later stages, indicating lactate accumulation in the TME or TIME (Figure 3) [87]. Lactate accumulation exerts immunosuppressive action by different mechanisms, including acidity and immune-cell apoptosis/death (Figure 3). Details of immunometabolic reprogramming responsible for the immunosuppressive TIME are discussed elsewhere by the authors [47].

An immunosuppressive TIME supports CC growth and metastasis. High levels of the immunosuppressive cytokines IL-10 and IL-13 are associated with cervical SCC (Figure 3). Increased levels of IFN-γ and IL-12p70 have antitumor action and are associated with decreased cervical SCC and grade incidence [88]. High IL-10 levels correlate with amplified arginase activity, decreasing L-arginine levels but increasing L-Arg metabolite levels, which is crucial to tumor growth [88,89,90,91]. HPV+ women, with either precancerous cervical lesions or CC, have elevated arginase levels, indicating persistent immunosuppression and a tumor-supportive immune environment [92]. Furthermore, indoleamine 2,3-dioxygenase (IDO) and tryptophan 2,3-dioxygenase (TDO) are also critical players in CC immunopathogenesis [93,94]. TDO catabolizes tryptophan (Trp) to kynurenine (Kyn), a binding ligand for the aryl hydrocarbon receptor (AHR), which is involved in multiple bioregulatory processes contributing to CC progression [95]. Kyn metabolites are critical to generating an immunosuppressive microenvironment, including the generation of tolerogenic DCs (tDCs), which suppress potent antitumor T cell-mediated immunity (Figure 3). IFN-γ also promotes IDO expression. IFN-γ and Kyn induce autophagy in CC cells in vitro but this has not been replicated in vivo [96].

TDO expression in leukocytes surrounding intraepithelial or invasive CC lesions is critical in CC progression [93]. TDO is a critical prognostic oncolytic biomarker, as its overexpression correlates with poor overall and progression-free survival [97]. Mechanistically, TDO catabolizes Trp to Kyn, a binding ligand for the aryl hydrocarbon receptor (AHR), which is involved in multiple bioregulatory processes contributing to CC progression [98,99]. Details of the AHR pathway and its role in cancer progression are beyond the scope of this review but are extensively discussed elsewhere [100,101]. CAC has a lower TGF-β level and activity than CC SCC [102]. Hence, HPV escapes the host immune response (immunosurveillance) to establish the infection and grows inside basal/suprabasal keratinocytes without their transformation. However, its growth and multiplication in epithelial cells transform them into neoplastic cells and generate different oncoproteins. These oncoproteins also suppress antitumor immunity. The continuous growth of tumor cells develops a nutrient-depleted microenvironment to support an immunosuppressive TIME.

## 4. Factors Regulating CC Immunosuppressive TIME

Immunosuppressive TIME is key for tumor growth, progression, and metastasis [47]. The cancer and immune-cell metabolites and differentially released factors promote the development of the immunosuppressive TIME [47,103,104,105]. The CC immunosuppressive TIME is regulated by different regulating factors, including the HPV E6 and E7 proteins, as discussed earlier which helps in the maintenance of the malignant phenotype of infected cells and explains the absence of antigen loss in HPV-associated CC [106]. CC cells show increased aerobic glycolysis that causes lactate accumulation in the CC TIME, which blocks the antitumor activity of local and infiltrated immune cells, including the plasmacytoid DCs (pDCs) and central memory T cells [47,107].

Furthermore, tumor or immune-cell-derived regulating factors, including FoxP3 (forkhead box protein P3), CCL22/CCR4, OX40L/OX40 (tumor necrosis factor superfamily member 4/tumor necrosis factor receptor superfamily member 4), and SMAD3 (SMAD family member 3) also determine the maintenance of the immunosuppressive CC TIME (Figure 3) [108]. For example, FoxP3, CCL22, and CCR4 overexpression and SMAD3 downregulation in the CC support the immunosuppressive TIME. Furthermore, CXCR3 expression also determines the CC TIME characteristic, as a decreased CXCR3 expression is associated with low M1 macrophages, activated memory CD4^+^ T cells, and CD8^+^ T cells [109]. However, CD163 expressing M2 macrophages are elevated in the CC TIME, which suppress the programmed cell-death protein 1 (PD-1 or CD279)/programmed-death ligand 1 (PD-L1 or CD274) blockers efficacy [110,111]. It is noteworthy that CC patients with decreased CXCR3 levels die soon, as compared to patients with higher CXCR3 expression due to the lack of antitumor response of CXCR3-signaling CD8^+^ T cells in the TIME.

PD-1/PD-L1 overexpression (T cells, DCs, and macrophages) is well correlated with high-risk HPV infection and its progression to the CC with increased mortality by supporting the immunosuppressive TIME (Figure 3) [110,112,113,114,115,116,117]. Cytotoxic T-lymphocyte-associated protein 4 (CTLA-4, another immune checkpoint) overexpression is also associated with CC immunosuppressive TIME (Figure 3) by increasing regulatory T cells’ (T_regs_) and conventional T cells’ antitumor function by regulating CD28 signaling that impacts their interaction with CD80 (B7.1) and CD86 (B7.2) [118,119]. Increased CTLA4 levels also correlate with IL-1β expression CC as IL-1β increases the signal transduction of the CTLA4; therefore, targeting the IL-1β–CTLA4 axis may help to overcome the CC immunosuppressive TIME, as seen in colon cancer [118,120]. Cystatin 7 (CST7) in T cells is also downregulated in the CC patients which induces dysfunctional antitumor CD4^+^ and CD8^+^ T cell immunity [118,121]. These dysfunctional CD8^+^ T cells comprise the major T-cell population in the CC TIME. ERBB3 (Erb-B2 Receptor Tyrosine Kinase 3) or HER3 or human epidermal growth factor receptor 3 (EGF3) methylation in CC also supports immunosuppressive TIME by regulating different chemokines (CXCL9, CXCL5, CXCL13, CXCL11, CCL19, CCL18, CCL21, and CCL22) and tumor-immune lymphocyte (TIL) infiltration and expression of different immune checkpoints, including T-cell immunoreceptor with Ig and ITIM Domains (TIGIT), CTLA4, and lymphocyte activation gene 3 (LAG3) [122]. Thus, several regulatory factors support the development of immunosuppressive CC TIME, which is further maintained by the polarization of local and infiltrated immune cells to protumor immune cells.

## 5. Immune Cell Populations in the CC TIME

The immune response plays a crucial role in detecting and preventing the development of CC. However, factors such as age, prior or repeated human papillomavirus (HPV) infection, changes in the microbiota of the reproductive tract, and lifestyle choices can lead to immune dysregulation and increase the risk of CC [123,124,125,126]. In addition, the composition of immune cells also varies in precancerous lesions and stages of CC, highlighting the importance of the TIME in CC progression.

One of the critical components of the TIME is tumor-infiltrating lymphocytes (TILs), which are altered in CC and can contribute to tumor growth. Consistent with other cancers, TILs are altered in CC, thus contributing to tumor growth and advancement. Individuals with high immune expression have a more robust CC prognostic outlook than patients with less active CC TIME [71]. Notably, as many as 60% of TILs in tumors comprise tumor-specific T-cells with a high aptitude for tumor eradication [127]. In the following section, we will explore the involvement of different immune cells in the CC immunosuppressive TIME as understanding TIME plays a critical role in developing effective cancer immunotherapies [128,129].

### 5.1. Tumor-Associated Macrophages (TAMs)

Tumor-associated macrophages (TAMs) are another crucial component of the TIME, and their levels can influence cancer prognosis depending on their diversity as determined through single-cell omics [130,131]. For example, they help in neoangiogenesis which helps in tumor growth, survival, and metastasis by releasing angiogeninc factors, including vascular endothelial growth factor (VEGF) and creating a tumor-promoting immunosuppressive environment (Figure 3). There are two subgroups of macrophages, M1 and M2, with different roles in cancer progression [130,132]. M1 macrophages may be less detrimental to cancer prognosis, as they are more associated with phagocytosis and antitumor inflammation reactions, while M2 macrophages exhibit immunosuppressive, tumor-promoting activities by secreting different molecules, including IL-10, TGF-β, prostaglandin E2 (PGE2, due to the overactivation of cyclo-oxygenase II or COX-II enzyme) (Figure 3) [130,133]. These immunosuppressive molecules stimulate Th2 immune response and promote T_reg_ polarization and function along with promoting the polarization and function of other immunosuppressive immune cells discussed later (Figure 3) [130] High-stage intraepithelial lesions are more likely to have M1 macrophages, while M2 macrophages are more common in tumors (Figure 1B) [134]. Elevated M2 levels in the TIME can create an immunosuppressive environment by supporting the generation and recruitment of myeloid-derived suppressor cells (MDSC) and regulatory T-cells (T_regs_) generation and recruitment (Figure 1B and Figure 3) [130,135] while suppressing antitumor cytotoxic NK cells [136]. The increased CD163^+^M2 macrophages in the CC TIME are highly associated with overexpression of PD-L1 that supports immunosuppression through T-cell exhaustion and suppresses the PD-1/PD-L1 blockers’ efficacy, as discussed previously [137].

Understanding the CC TIME can help mitigate the detrimental effects of TAMs on prognosis. For instance, blocking the expression of transmembrane protein neuropilin-1 (NRP1) in M2 macrophages can prevent M2 polarization and recruitment [138]. Additionally, radiotherapy can repolarize tumor-promoting M2 macrophages to tumor-killing M1 macrophages [139]. Mitigating M2 prevalence in the TIME could be instrumental in stimulating immune responses to target CC [140]. Developing chimeric antigen receptor (CAR)-macrophages also has excellent potential in CC immunotherapy to overcome the immunosuppressive TIME, a significant hurdle in successful cancer clearance [141,142].

### 5.2. Neutrophils in the CC TIME

Neutrophils are immune cells that can either suppress or enhance tumor growth, depending on their function in the TIME [143,144,145]. In humans, neutrophils make up 50–70% of all circulating leukocytes, but this percentage may increase in tumors [146]. An increase in neutrophil count is associated with decreased overall survival for patients with CC. Overall, CC-patient survival decreases with the increase in absolute neutrophil count (≥6187/mm^3^) (Figure 1B) [147]. For example, an elevated CD66b^+^ tumor-associated neutrophil (TAN) count is also linked to shorter recurrence-free survival in CC due to increased neutrophil extracellular traps (NETs) [148], contributing to tumor proliferation and metastasis (Figure 3) [149]. The neutrophil-mediated IL-17 release in patients with squamous CC decreases their survival by creating a tumor-promoting TIME at early tumorigenesis [150].

The neutrophil-lymphocyte ratio (NLR) can be an independent prognostic factor in CC, with an NLR ≥ 3.6 indicating a poor overall response rate and survival [151]. NLR can also help predict the CC therapeutic response. For example, patients with an NLR of less than eight have a 57% chance of one-year survival following PD-1/PD-L1 inhibitors, while individuals with an NLR of eight or below have only a 27% chance of one-year survival [152]. A possible explanation of this trend is that neutrophils are associated with elevated cytokine secretions, thus enhancing tumor growth and metastasis [151]. Thus, an overaccumulation of tumor-supportive neutrophils further supports the immunosuppressive TIME. Therefore, therapeutic exploitation of TANs is critical in the successful tumor immunotherapy [153,154,155,156].

### 5.3. MDSCs in the CC TIME

Approaches targeting MDSCs in the CC TIME are critical to overcoming the immunosuppressive TIME and increasing the efficacy of existing immunotherapies [153,154,155,156]. Three types of MDSCs including monocytic, polymorphonuclear, and early-stage cells exert potent immunosuppressive action [157,158,159]. In addition, MDSCs secrete tumor-promoting arginase that generates tumor-promoting immunosuppressive polyamines in the CC TIME [157,160]. However, MDSCs activity in CC is governed by many aspects of the TIME, including immune and nonimmune cells and released factors. For example, growth factor granulocyte-colony stimulating factor (G-CSF) increases MDSCs’ number in CC (Figure 1B and Figure 3). Elevated G-CSF can induce tumor-related leukocytosis (TRL), a condition found in patients with advanced cancer [161]. Specifically, patients with CC with TRL are at greater risk for metastasis and are low responders to radiotherapy [162,163]. Given the connection and detrimental effect of MDSCs and G-CSF levels, future CC therapies should focus on alternative therapies to mitigate these levels. Novel immunotherapeutic approaches could focus on mitigating MDSCs and controlling G-CSF levels.

Furthermore, T cells also affect MDSCs’ function in TIME and vice-versa [164]. The combined activity of m-MDSCs and mucosal-associated invariant T (MAIT)-cells may be associated with CC progression (Figure 1B) [165]. All-trans retinoic acid (ATRA, a vitamin A-derivative) influences MDSCs maturation and eliminates their immunosuppressive activity. ATRA treatment decreases MDSC accumulation in BALB/C mice with CC and increases antitumor cytotoxic CD8^+^ T cells [166]. Combined ATRA and anti-PD-L1 therapies may be promising approaches to CC cancer immunotherapy, as this approach delays tumor growth and increases antitumor T-cells, IFN-γ, and TNF-α levels [166]. Thus, approaches targeting MDSCs in the CC TIME are critical to overcoming the immunosuppressive TIME and increasing the efficacy of existing immune checkpoint inhibitors (ICIs) and other immunotherapies.

### 5.4. MAIT Cells in the CC TIME

MAIT cells are unique T cells found in the body’s peripheral blood, liver, and mucosal surfaces, including the cervix [167]. They produce cytokines such as IFN-γ and IL-17, which are crucial in fighting pathogens such as bacteria, viruses, and fungi. They also regulate inflammatory responses and contribute to immune-mediated diseases [167,168,169,170]. MAIT cells are essential in different cancers and have potential in immunotherapy [171,172,173,174,175,176,177].

In patients with CC, there is a decrease in the number of MAIT cells in circulation, which is associated with poor progression-free survival [178]. The number of CD4-CD8-PD1^+^ MAIT (DN or double negative) cells in the peripheral circulation of patients with CC is directly related to disease severity [165]. The decrease in peripheral DN MAIT cells indicates that they might have migrated to the cancer tissue, further supporting the growth of the tumor [165]. Further investigation into the number and types of MAIT cells in CC biopsies and animal studies is necessary to understand this critical area of tumor immunology.

MAIT cells in the tumor microenvironment (TME) can either hinder or aid the antitumor activity of NK cells. While MAIT cell accumulation can contribute to tumor growth and spread, activating these cells can enhance their antitumor action by activating NK cells [179]. In cancer immunotherapy, inhibiting MHC class I-related protein 1 (MR-1) on cancer cells can be used to develop MAIT-cells-based treatments. The interaction with MR-1 expressed on tumor cells and TIME MAIT cells activate them to release IL-17A, suppressing cytotoxic T and NK cells. Thus, blocking MR-1 on cancer cells may help design MAIT-cells-based cancer immunotherapy. Additionally, combining a synthetic riboflavin synthesis pathway-derived antigen 5-OP-RU [5-(2-oxopropylideneamino)-6-D-ribitylaminouracil] and the CpG (a TLR9 agonist) can boost the antitumor immune response of TIME MAIT cells as indicated by the increased levels of CD69 expression, pronounced effector memory phenotype, and upregulation of effector molecules, including IFN-γ, granzyme B (GrB), and perforin [180]. Interestingly, 5-OP-RU and TLR9 agonist combination work independently of MHC class I related-1 molecule (MR1) expression in tumor cells. Reprogramming and redifferentiating TIME MAIT cells are also promising approaches for cancer immunotherapy. Therefore, exploring and targeting TIME MAIT cells in CC is a new and innovative cellular immunotherapy strategy.

### 5.5. Mast Cells in the CC TIME

Mast cells are critical innate immune cells with different immune and inflammation regulatory functions [181,182]. Initially, they were only associated with allergic reactions/diseases. However, recent advancements in immunology have established them as potent immunoregulatory cells that perform various immune functions, including maintaining immune homeostasis [181,182,183,184]. Mast cells play a critical role in the TIME of many cancers and their effect on promoting or inhibiting tumors depends on the type and stage of cancer [185,186]. Mast cells (tryptase-positive and tryptase/chymase-positive) are also found in the normal human cervix, and their number increases in benign inflammatory conditions [187,188,189]. A recent study reported a widespread distribution of mast cells in CC tissues and patients with low mast-cell density in their TIME had better overall survival rates [190]. Mast cells promote tumor growth by supporting neoangiogenesis and creating an immunosuppressive TIME. Specific mast-cell mediators, including histamine, TNF-α, and cannabinoids, also contribute to CC cell invasion and metastasis (Figure 3) [183,184,185,186,191]. In addition, mast-cell infiltration in the TIME increases the tumor’s resistance to anti-PD-1 immune checkpoint blockers [192,193]. Hence, targeting mast cells and their mediators in the CC TIME may inhibit CC growth and metastasis by inhibiting neoangiogenesis and immunosuppressive events that support CC metastasis.

### 5.6. Eosinophils in the CC TIME

Eosinophils are critical innate immune cells that are present in low numbers in blood but are present in higher numbers at mucosal surfaces [194,195,196]. They play a critical role in antimicrobial immunity, allergies, and tumor immunity [197,198,199]. For example, eosinophils work with DCs and T cells to produce inflammatory and adaptive immune responses [200]. A patient’s eosinophil count can predict the immune response in cervical SCC, and high levels of eosinophils can result in poor survival (Figure 1B) [201,202]. Hypoxic conditions influence the function of EOs during cancer progression. The prevalence of eosinophils increases with CC progression, and hypoxic conditions also influence their function [193,203]. Thymic stromal lymphopoietin (TSLP) stimulates CC growth [204] and regulates eosinophil activity in the hypoxic CC TME. The increased TSLP upregulates CCL17 production [193,204], which over-recruits eosinophils (Figure 3). In addition, TSLP promotes CC progression by promoting the immunosuppressive Th2 immune response (Figure 3). Eosinophils can create an immunosuppressive TIME by releasing Th2 cytokines and suppressing NK and T-cell functions in the CC (Figure 3) [205,206].

### 5.7. DCs in the CC TIME

DCs are an essential immune system component that can either enhance or suppress tumor response [200,201,202,203,204,205,206,207]. The different types of DCs, including conventional DCs (cDC1s and cDC2s), plasmacytoid DCs (pDCs), and mature DCs, express varying levels of costimulatory molecules and immune checkpoints, depending on the type and stage of the tumor [208,209]. For instance, cDC1s typically do not express PD-L1 and immunoglobulin-like transcript 2 (ILT2) under normal conditions. However, during tumor progression, they may express high T-cell immunoglobulin and mucin-domain containing-3 (TIM-3), a unique immune-checkpoint repertoire [209]. TIM-3 interacts with galectin-9, a C-type lectin, to stimulate antitumor function in innate immune cells, such as DCs, NK cells, and macrophages, by activating proinflammatory signaling pathways, including PI3K-mammalian target of rapamycin (mTOR) and hypoxia-inducible factor-1 (HIF-1) signaling without inducing apoptosis [203,204,205,206,207,208].

The decrease of galectin-9 in CC (CIN and SCC) patients increases its severity, whereas its increase is associated with a better prognosis regarding the overall survival [209,210]. However, severe CC cases (advanced stage IV) have an increased systemic galectin-9 level, indicating that circulating galectin-9 via TIM-3 interaction in systemic Th1 and CD8^+^ T cells induces their apoptosis and impairs their infiltration in the CC TME [211]. Furthermore, in HPV-associated patients with CC, circulating CD4^+^ and CD8^+^ T cells overexpress TIM-3, supporting that increased circulating galectin-9, including monocyte-specific galectin-9 in patients with CC, induces Th1 and CD8^+^ T cell apoptosis to suppress systemic T-cell-dependent antitumor immunity [212]. Thus, increased circulating TIM-3 expression of T cells and galectin-9 in patients with CC is associated with poor CC prognosis. In addition, increased TIM-3 expression in T_regs_ via galectin-9 interaction increases the immunosuppressive function (IL-10 and TGF-β release) in patients with CC [212,213]. Notably, the systemic galectin-9 level is independent of the local CC TME. Hence, local CC TME galectin-9 decreases the CC severity and improves overall patient survival, whereas systemic galectin-9 is associated with increased CC severity. Carcinoembryonic antigen-related cell adhesion molecule 1 (CEACAM-1) is an adhesion molecule that also serves as a heterophilic ligand for TIM-3 expressed on T cells [214]. In severe or high-grade squamous intraepithelial lesions (SIL) of patients with CC, the increased CEACAM-1 and TIM-3 interaction further suppresses the antitumor activity of CD4^+^ and CD8^+^ T cells [215]. Furthermore, CC cells overexpress HMGB1 and serve as a prognostic indicator and a potential biomarker, suppressing antitumor T-cell function via interacting with TIM-3 [216,217,218,219,220].

cDC1s are critical for antitumor cytotoxic T cells and decrease T_regs_ via secreting the cGAS/STING signaling pathway-dependent type 1 IFNs [221]. The cDC1s’ decrease in the CAC TIME is associated with poor patient survival due to impaired T-cell-mediated antitumor immunity (Figure 1B and Figure 3) [222]. Although cDC1s highly express TIM-3, their decrease in severe CC cases indicates that they could not exert their antitumor action. Therefore, it should be interesting to investigate whether they die or transform to tumor-supportive tolerogenic DCs in the immunosuppressive CC TIME. The increased HMGB1 level in the CC TIME is associated with promoting pDCs to tDCs that further supports immunosuppressive TIME for the CC progression (Figure 2 and Figure 3) [223]. Furthermore, low cDC1 chemo-attractive chemokines in the CC TIME support the immunosuppressive niche. For example, NK cells in the TIME support cDC1 infiltration by releasing XCL1 and CCL5, and NK cells lose this function in the presence of PGE2 [224]. Overexpressed PGE2 in the CC TIME suppresses NK cell-mediated release of cDC1 chemokines to dysregulate the NK cell–cDC1–chemokine axis [225,226]. Thus, HMGB1 and PGE2 in the CC TIME suppress antitumor T cell, NK cell, and DC function, supporting CC growth and metastasis (Figure 3).

HPV-VLP vaccination stimulates DC and NK cell crosstalk to exert the antitumor activity in patients with CC, as indicated by the CD69 (an activation marker) and HLA-DR upregulation on DCs and increased NKCC and IFN-γ release [227,228,229]. DC-derived exosome vaccines also induce antitumor cytotoxic CD8^+^ T cell activity, proliferation, and IFN-γ secretion [230]. Immunotherapeutics work effectively under the right immunological conditions. For example, the synthetic dsRNA viral analog Poly I: C (polyinosinic: polycytidylic acid) vaccine is a promising CC vaccine. Poly I: C vaccine induces receptor-interacting protein kinase 3 (RIPK3) signaling for its direct cytotoxicity on tumor cells. The CC-cell necroptosis induces IL-1α release, which activates DC-mediated IL-12 production, critical for an antitumor immune response [231]. Thus, increasing cDC1s in CAC patients will increase their survival via increasing antitumor immunity. Further studies in this direction are critical to designing DC-specific vaccines for CC.

### 5.8. NK Cells in the CC TIME

NK cells are potent antitumor innate immune cells categorized as type 1 innate lymphoid cells (ILCs) [210]. Their role in antitumor immunity, including uterine cancer, has been discussed elsewhere [211,212]. However, in CC, the number and function of NK cells are reduced due to various cellular processes (Figure 1B). For example, CD3^+^CD56^+^ NK cell infiltration increases at early CC stages, which decreases as cancer progresses to advanced stages due to higher TGF-β1 in the tumor. TGF-β1 inhibits natural-killer group 2D (NKG2D), CD16, and Ki67 receptor function [213]. The decreased NK cell number in CC TIME is associated with HLA-I downregulation, potentially due to upregulated immunosuppressive cytokines, including IL-10, IL-13, and TGF-β [214], which inhibit NK-cell function. Patients with CC have HLA-E (a major histocompatibility (MHC) class I molecules involved in the NK-cell recognition pathway) overexpression, which is not well-associated with the prognostic outcome, potentially due to a high volume of exhausted or apoptotic CD8^+^ T cells [215]. However, elevated HLA-E expression in patients with CAC improves survival [216]. The decreased NK-cell number in the CC TIME further supports the decrease in potent antitumor cDC1s in patients with advanced CC. Therefore, the strategies to design NK-cell-based immunotherapies to target CC will be an exciting area to explore.

### 5.9. T Cells in the CC TIME

The type and concentration of T-cells in a person’s immune system can provide insight into their immune response and prognosis for CC. T cells infiltrate CC tumors, but the CD4^+^: CD8^+^ differs from that in the peripheral blood [217] and lower CD4/CD8 ratios are associated with faster tumor growth and lymph-node metastasis [218]. For example, healthy women have a CD4/CD8 ratio of 1.42 [219] but this number decreases to 0.6 and 1.17 in women with fatal and nonfatal CC, respectively [220]. These trends can be explained mechanistically, as CD4^+^ T cells activate the cytotoxic CD8^+^ T cells, and T_regs_ accumulate near advanced tumors, inhibiting antitumor immune activity. Th1 cells’ number and function also alter CC carcinogenesis (Figure 1B). For example, Th1 levels increase from low to high-grade squamous intraepithelial lesions but deplete from high-grade squamous intraepithelial lesions to SCC. In contrast, Th2 levels deplete from low- to high-grade squamous intraepithelial lesions [221]. Th2 and Th17 populations increase, and Th1 levels are depleted in CIN and CC, which supports that a shift in these cell populations starts prior to CC formation and, thus, contributes to CIN progressing into CC (Figure 1B) [222]. Th1 dominance is critical to antitumor immunity and contributes to immune memory, forming tumor-specific cytotoxic T-lymphocytes (CTLs) [223], further suggesting that Th1 depletion is crucial to CIN progression into CC.

HPV infections preceding CC development contribute to T-cell alterations. For example, CD4^+^ T-cells specific to HPV^+^ patients with CC can suppress T-cell proliferation and alter their function [223]. The ratio of cell types changes not only with cancer status but also with HPV status. For example, CD8^+^ T cells are more prevalent than CD4^+^ in the epithelial layer of an HPV^+^ normal cervix, but this becomes less prominent with an increasing CIN grade [225]. The CD4/CD8 ratio, as well as the quantity of CD4^+^ T cells, are indicative of CC survival in HPV^+^ individuals. Overall, a lower number of T_regs_ is detrimental to five-year survival but, more specifically, individuals with lower CD4/CD8 ratios have higher mortality rates than those with higher ratios [220]. Women undergoing neoadjuvant chemotherapy have more remarkable survival if they have higher CD4/CD8 ratios before their third round of treatment [226]. Furthermore, neoadjuvant chemotherapy increases CD4, CD8, CD20, and CD56 signals, most prominently in good responders, indicating the activation of antitumor Th1, cytotoxic T, B, and NK cells [227]. Therefore, the immunoactive TIME in good responders is crucial to support locoregional stimulation of antitumor immunity during neoadjuvant chemotherapy. Hence, neoadjuvant chemotherapy can be combined with ICIs in patients with CC to stimulate antitumor immunity.

Specific gene-expression profiles and ligands can significantly impact T_regs_ within the CC TIME. For example, Foxp3 and V-domain immunoglobulin suppressor of T-cell activation (VISTA) significantly correlate with CC prognosis, exhibiting higher expression in CC than in CIN or chronic cervicitis. Specifically, patients with double-negative (Foxp3 and VISTA) tumors show the best prognosis, while double-positive patients show the worst prognosis [228]. Foxp3 levels are also higher in patients with lymph node metastasis than those without metastasis [229]. Foxp3 levels are also higher in patients with CC with lymph-node metastasis than those without metastasis [229]. The increased FoxP3 expression in Th1 cells to transform them to T_regs_ occurs due to the intracellular STING activation in these T cells [230]. The intrinsic STING activation in T cells induces TANK binding kinase-1-interferon regulatory factor (TBK1-IRF3)-mediated mothers against decapentaplegic homolog 3 or SMAD3 and signal transducer and activator of transcription 5 (STAT5) phosphorylation independent of IFN-β to induce FoxP3 activation and their transformation to T_regs_. In CC TIME, tumor-derived exosomes with TGF-β, cGAS, and 2′-3′-cGAMP activate STING signaling in tumor-infiltrated T cells to promote induced-Treg (iTreg) expansion [230].

Understanding T-cell-specific TIL activity changes is critical to designing better T-cell-based immunotherapeutics specific to CC type. Patients with CC have better effector T-cell infiltration than adenocarcinoma patients, with elevated CD45^+^ and CD3^+^ levels, T_regs_, and PD-1 and TIM-3 immune checkpoints. These changes are prognostically significant and may indicate immunotherapeutic responses, as increases in CD3^+^ densities can decrease the death and relapse risk [231]. Therefore, immunotherapeutics that work well for SCC may not work as well in adenocarcinoma. Nevertheless, tumor-specific T cells are ideal candidates for personalized, adaptive immunotherapy. TILs from individual patients are primed for specific tumors and are immediately ready to return after infusion [127].

Under conditions that support oxidative phosphorylation (OXPHOS), Th17 cells have increased persistence and can decrease tumor growth in vivo [232]. In squamous CC TIME, an increased presence of Th17 cells has been associated with improved patient survival due to their anticancer effects [150,233]. The decreased presence of galectin-9 in the CC TIME promotes the development of Th17 cells, as galectin-9 interaction with TIM-3 induces apoptosis in mature Th17 cells [234]. However, Th17 cells in CAC are detrimental to the patient and can increase tumor growth and severity and contribute to CAC relapse after tumor removal [235]. The infiltration of Th17 cells in the CC TIME is facilitated by CCL20, which binds to overexpressed CCR6 [236]. Therefore, it would be interesting to explore the immunometabolic reprogramming of Th17 cells that govern their anti- and protumor functions in squamous and adenocarcinoma patients with CC to develop immunometabolism-based Th17 cell-directed immunotherapies.

### 5.10. B Cells in the CC TIME

B-cells play a crucial role in regulating the immune system by producing antibodies and releasing cytokines [237]. Studies on mice have shown that reducing the B-cell count can boost the body’s antitumor response by lowering IL-10 production and increasing IFN-γ levels from CD8^+^ T-cells and NK cells [238]. However, a subset of B-cells called Bregs can hinder cytokine secretion and counteract the antitumor response of other immune cells [239]. Understanding the role of B-cells in cancer immunity is critical, especially for HPV-associated cancers, which have shown conflicting results in human patients. For example, B-cells exert a vital antitumor role in HPV^+^ patients with CC [240]. Therefore, a deeper understanding of a patient’s cancer type is required in treatment.

Researchers are exploring the potential of B-cell-targeted immunotherapy in cancer treatment. For instance, PD-1 blockade and radiotherapy have proven effective in increasing memory B-cells, antigen-specific B-cells, and plasma cells in HPV-associated cancers [240]. However, Bregs have been found to inhibit CD8^+^ T-cell cytotoxicity in CC, leading to lower prognostic outcomes in individuals with low CD4/CD8 ratios [241]. There is still much to learn about the role of B-cells and Bregs in CC and their modulation of immunotherapy.

## 6. Targeting the CC TIME

Currently, different preventive and therapeutic approaches (which also have severe adverse reactions) are available for CC (Table 1), but we do not have effective immunotherapeutics to treat or target CC [242,243,244,245]. Targeting the TIME is a promising approach for cancer-specific immunotherapy, including CC. Researchers are studying the immunoregulatory factors of TIME to develop effective CC immunotherapies. For example, treating cervical epithelial cells with poly (dA:dT, a synthetic dsDNA analog) has shown potential in activating antiviral immunity and increasing CD8^+^ T-cell and DC populations to clear the tumor. This treatment induces the expression of different IFNs and associated IFN-stimulate genes (ISGs, including ISG-15, ISG-56), 2′-5′-oligoadenylate synthetase 1 (OAS1), OAS2, myxovirus resistance protein A (MxA), MxB, virus inhibitory protein, endoplasmic reticulum-associated, IFN-inducible (VIPERIN), and guanylate-binding protein 1 (GBP5)-dependent antiviral immunity by the activating retinoic acid-inducible gene I (RIG-1)-like receptor (RLR) signaling pathway [246,247]. This experimental approach has great potential to target HR-HPV-associated CC. CEC-specific cGAS/STING signaling activation by ADU-S100 (S100) promotes its antiviral and antitumor activity by acutely releasing type 1 IFNs and NF-κB-dependent proinflammatory cytokines, such as IL-6 and TNF-α [248]. In addition, ADU-S100 increases the cytotoxic CD8^+^ T cell and CD103^+^DC population to clear the tumor in vivo [248,249]. CD103^+^DCs also increase the efficacy of ICIs in TIME [250,251]. Therefore, HR-HPV targeted therapeutic vaccines could prove advantageous for CC. For example, NK cell-based therapy against the upregulation of HPV-VLPs in CC could be an exciting avenue to explore, as they appear to stimulate FRT NK cells [252]. Thus, HPV-targeted therapeutic vaccines and NK cell-based therapy against HPV-VLPs upregulation in CC are exciting avenues to explore.

Numerous ICIs are currently undergoing clinical trials, and some have shown promising results in combination with existing treatments [263,264,265,266,267,268,269,270,271,272,273,274,275,276,277,278,279,280,281,282]. For example, pembrolizumab increases the efficacy of existing chemotherapies in treating PD-L1-positive chemotherapy-resistant metastatic CC [264]. Cotreatment with pembrolizumab and the GX-188E vaccine is safe for HR-HPV infections and associated advanced CC [265]. Furthermore, HPV nanovaccine combination with laser therapy inhibits the CC progression by activating T-cells and inducing DC maturation [266]. However, translating experimental findings to humans requires a better understanding of the TIME in CC. For instance, radiotherapy may decrease the immune-cell population and their cytotoxic potential while increasing PD-1 capacity in CD4^+^ T cells. This trial has also uncovered further immune dysregulation due to elevated monocyte and MDSC levels [267], which can be detrimental to cancer immunotherapy. 

Adoptive cell therapies (ACTs, including tumor-infiltrating T lymphocytes (TILs) and CAR-T cells) comprise a form of cancer immunotherapy, including ICIs and vaccines [268,269,270]. The details of engineered T cells with a potential to use in cancer immunotherapy have been discussed elsewhere [271]. Autologous TILs are ex vivo expanded tumor infiltrating T cells rescued from the tumor tissue and are transplanted back to the patient following lymphodepletion that has been mostly evaluated in metastatic skin melanoma and recurrent/metastatic CC of the cervix successfully as compared to other solid cancers [272,273]. Hence, autologous TILs (LN-145) offer CC patients a safe and viable therapeutic approach warranting further investigations [273]. TILs and their use in different solid tumors are discussed in detail elsewhere [274,275,276]. Further advances in cancer immunology have led to the development of CAR-T cells’-based cancer immunotherapies. CAR-T cell therapies have shown effectiveness against certain types of cancer, such as leukemia, but have limited efficacy against solid tumors [277]. However, researchers are currently investigating modified therapies, such as Mesothelin (MESO) CAR-T cell therapy, which has shown promising results in detecting MESO in SiHa cell lines. This approach has the potential to increase antitumor cytokine production and improve outcomes [278].

One challenge to CAR-T cell therapy in solid tumors is immune checkpoints, including the PD-1–PD-L1 axis, which can decrease efficacy [277]. However, the genetically modified CAR-T-PD1 approach has shown increased efficacy in experimental studies with DCs. This approach increases antitumor cytotoxic activity, suppresses tumor growth, and elevates IL-2, IFN-γ, and TNF-α secretions, ultimately improving survival [277]. Another approach under investigation is CAR-T cell therapy using NKG2D, which has shown excellent tumor clearance with low toxicity in CC cells [279]. These studies suggest CAR-T cell therapy can effectively treat CC when engineered to consider the unique CC TIME. Furthermore, T-cell receptor (TCR)-engineered/modified effector T (TCR-T) cells are another class of adoptive T-cell therapy, which have naturally occurring or minimally modified TCRs to develop T-cell-based immunotherapy for cancers [280]. These TCR-T cells recognize tumor-specific epitopes presented by major-histocompatibility complex (MHC) molecules expressed on cancer cells which gives them the advantage of potentially broader application. This is because of the presence of larger tumor-specific sequences within a cell and presented in the MHC than tumor-specific proteins on the surface [280]. TCR-T cells may have a higher potency than CAR-T cells for solid cancers, including CC, due to the MHC presentation of intracellular antigens specifically present in cancer cells. The details of TCR-T for solid tumors are discussed elsewhere [280,281]. Additionally, adeno-associated virus (AAV)-based gene therapy, including the delivery of oncolytic agents/drugs, has shown potential for personalized medicine or immunotherapy based on the HR-HPV type and patient genetic and immune status [282].

## 7. Future Perspective and Conclusions

Over the past 10–20 years, tumor immunology has made significant progress thanks to advancements in immunology, which have helped understand the growth, development, and spread of cancer cells. This progress has led to the discovery of ICIs as cancer immunotherapy by James P. Allison and Tasuku Honjo, which was awarded the Nobel Prize in Physiology and Medicine in 2018. In addition, researchers have identified that HR-HPVs are primarily responsible for CC in humans [283,284,285]. Understanding the immunosuppressive environment of CC is crucial for developing novel immunotherapies to advance treatment. A thorough understanding of the genetics and immune cells involved in CC can help predict therapeutic responses and allow for patient-specific approaches. Various immune genes have been used to predict changes in the immune microenvironment, which serve as indicators for immunotherapeutic response and survival [286]. For example, patients with high levels of CD8^+^ T cells in their tissues have shown more robust immunotherapy response rates [287]. Different immune cells, including LCs and MAIT cells, have great potential for developing cancer-specific immunotherapy [177,288,289]. In addition, HR-HPV CCs expressing viral L1 protein (major capsid protein) can be targeted by effective L1/tumor-specific CD4^+^ and CD8^+^ T cells or combined E7/L1 DC-based vaccines [290]. Furthermore, galectin-9 modifying/targeting strategies also have an excellent potential for modulating immunosuppressive CC TIME and developing novel immunotherapies [291,292,293]. Tumor-resident Mast cells are also emerging as novel immunotherapeutic targets for targeting the immunosuppressive TIME, which can be implied in patients with severe CC [294,295]. In conclusion, CC is a growing concern worldwide and it is crucial to understand its environment for future personalized immunotherapeutics to advance and to improve currently available therapies.

## Figures and Tables

**Table 1:** Currently available CC preventive vaccines and therapeutics, including monoclonal antibodies, therapeutic vaccines, and antibody–drug conjugates (ADCs).

CC Prevention Strategies	Target
Cervarix (Recombinant HPV bivalent vaccine, comprising HPV16 and HPV18 L1 virus-like particles (VLPs) formulated in ASO4 (alum combined with a TLR4 ligand, MPL (3-O-desacyl-4′-monophosphoryl lipid A) adjuvant) [244]. It is used in females aged between 10–25 years and is not available in the USA.	Prevents HPV-16 and -18-associated CC via inducing immunity, including anti-HPV-16 and -18 antibodies (IgG1)
2. Gardasil (Recombinant HPV quadrivalent vaccine, no longer available in the USA) [243] and Gardasil 9 (Recombinant HPV nonavalent vaccine, available in the USA, age of administration 9–45 years) [253]	Gardasil protects against low-risk HPV-6 and -11, which cause most genital warts, and against HR-HPV-16 and -18 for at least five years [243]. Gardasil 9 protects against infection with low-risk HPV types 6 and 11, which cause most genital warts, and against HR-HPV types 16, 18, 31, 33, 45, 52, and 58, responsible for different cancers by inducing the humoral antiviral immunity [253].
**Targeted drug therapies for CC**	
Bevacizumab (Avastin, Alymsys, Avastin, Mvasi, or Zirabev) is USFDA approved and used in combination with chemotherapies, including paclitaxel and either cisplatin or topotecan hydrochloride [242]	Targets human vascular endothelial growth factor (VEGF) to inhibit angiogenesis or neoangiogenesis in CC
2. Brivanib, currently under evaluation (phase II trial) to target advanced CC [242,254]	Targets VEGF and fibroblast growth factor receptor (FGFR)
3. Pembrolizumab (Keytruda), a USFDA-approved immune checkpoint inhibitor (ICI) [242]	Targets PD-1 on T cells to prevent their exhaustion that increases anticancer immunity to clear CC cells
4. Nivolumab, under phase II clinical trial. It has low antitumor activity and an acceptable safety profile in patients with persistent/recurrent CC [255]	Targets human PD-1
5. Ipilumab and Nivolumab combination in recurrent/metastatic CC patients [256]	Blocks PD-1–PD-L1 interaction to enhance the antitumor immunity
6. Cemiplimab, a second-line therapy for patients with persistent/recurrent CC [257]	Targets human PD-1 to block PD-1–PD-L1 interaction to prevent immune exhaustion
7. Tislelizumab, approved by China’s National Medical Products Administration and under USFDA review for different solid cancers. It has also shown beneficial antitumor activity and tolerable toxicity in patients with recurrent/metastatic CC [258]	Targets human PD-1 to inhibit PD-1–PD-L1 interaction and minimizes binding to Fcγ receptors to serve as an ICI.
8. Axalimogene filolisbac (ADXS11-001), a live, attenuated *Listeria monocytogenes* bacterial vector secreting HPV-16 E7 fused to listeriolysin O (LLO), a therapeutic vaccine in patients with recurrent/refractory CC patients [259,260]	Raises anti-HPV-16 cellular immunity, including cytotoxic T cell-mediated immune response
9. Tisotumab vedotin-tftv (Tivdak), An USFDA approved antibody–drug conjugate (ADC) for recurrent/metastatic CC [261,262]	This ADC delivers cytotoxic agent monomethyl auristatin E (MMAE) directly into tumor cells to target highly expressed tissue factor (TF) or conjugation factor III in CC

## Data Availability

Not applicable.

## Abbreviations

AAV	Adeno-associated virus
AC	Adenocarcinoma
ACTS	Adoptive T-cell therapies
AHR	Aryl hydrocarbon receptor
AIF	Apoptosis inducible factor
AIM-2	Absent in melanoma-2
AMP	Antimicrobial peptides
APC	Antigen-presenting cell
BECs	Basal epithelial cells
CAC	Cervical adenocarcinoma
CAR-T cell	Chimeric antigen receptor T cell
CC	Cervical cancer
CECs	Cervical epithelial cells
cGAMP	Cyclic GMP-AMP
cGAS	cGMP-AMP synthase
CIN	Cervical intraepithelial neoplasia
CLR	C-type lectin receptors
CMI	Cell-mediated immunity
CTL	Cytotoxic T lymphocyte
DNAM-1	DNAX accessory molecule-1
DGE	Differential gene expression
FoxP3	Forkhead box protein P3
FRT	Female reproductive tract
HIF	Hypoxia-inducible factor
HR-HPV	High-risk HPV
hTERT	Human telomeres reverse transcriptase
ICIs	Immune checkpoint inhibitors
IDO	Indolamine dioxygenase
LCs	Langerhans cells
MAIT	Mucosal-associated invariant T
MIP	Macrophage inflammatory protein
MUC	Mucin
NET	Neutrophil extracellular trap
NKCC	NK-cell-mediated toxicity
NKG2D	Natural killer group 2D
NLR	Neutrophil lymphocyte ratio
NOD	Nucleotide-binding oligomerization domain
PRR	Pattern-recognition receptor
RGS	Regulator of G protein signaling
RICK	RIP-like interacting CLARP kinase
SCC	Squamous cell carcinoma
SIL	Squamous intraepithelial lesion
STING	Stimulating interferon genes
TCR	T-cell receptor
TCR-T cells	TCR-engineered/modified effector T cells
TDO	Tryptophan dioxygenase
TIME	Tumor immune microenvironment
TME	Tumor microenvironment
TGF-β	Transforming growth factor beta
TSLP	Thymic stromal lymphopoietin
TZ	Transformational Zone
VISTA	V-domain immunoglobulin suppressor of T cell activation

## References

[1] Sung H., Ferlay J., Siegel R.L., Laversanne M., Soerjomataram I., Jemal A., Brey F. (2021). Global Cancer Statistics 2020: GLOBOCAN Estimates of Incidence and Mortality Worldwide for 36 Cancers in 185 Countries. CA A Cancer J. Clin..

[2] Tantengco O.A.G., Menon R. (2020). Contractile function of the cervix plays a role in normal and pathological pregnancy and parturition. Med. Hypotheses.

[3] Francoeur A.A., Liao C.-I., Casear M.A., Chan A., Kapp D.S., Cohen J.G., Salani R., Chan J.K. (2022). The increasing incidence of stage IV cervical cancer in the USA: What factors are related?. Int. J. Gynecol. Cancer.

[4] Gustafsson J.K., Johansson M.E.V. (2022). The role of goblet cells and mucus in intestinal homeostasis. Nat. Rev. Gastroenterol. Hepatol..

[5] Hasnain S.Z., Gallagher A.L., Grencis R.K., Thornton D.J. (2013). A new role for mucins in immunity: Insights from gastrointestinal nematode infection. Int. J. Biochem. Cell Biol..

[6] Johansson M.E., Hansson G.C. (2016). Immunological aspects of intestinal mucus and mucins. Nat. Rev. Immunol..

[7] Lee S.K., Kim C.J., Kim D.-J., Kang J.-H. (2015). Immune Cells in the Female Reproductive Tract. Immune Netw..

[8] Han L., Taub R., Jensen J.T. (2017). Cervical mucus and contraception: What we know and what we don’t. Contraception.

[9] Gipson I.K. (2001). Mucins of the human endocervix. Front. Biosci..

[10] Gipson I.K., Spurr-Michaud S., Moccia R., Zhan Q., Toribara N., Ho S.B., Gargiulo A.R., Hill J.A. (1999). MUC4 and MUC5B transcripts are the prevalent mucin messenger ribonucleic acids of the human endocervix. Biol. Reprod..

[11] Linden S.K., Sutton P., Karlsson N.G., Korolik V., McGuckin M.A. (2008). Mucins in the mucosal barrier to infection. Mucosal Immunol..

[12] Sheng Y.H., Hasnain S.Z. (2022). Mucus and Mucins: The Underappreciated Host Defence System. Front. Cell. Infect. Microbiol..

[13] Nasu K., Narahara H. (2010). Pattern Recognition via the Toll-Like Receptor System in the Human Female Genital Tract. Mediat. Inflamm..

[14] Hart K.M., Murphy A.J., Barrett K.T., Wira C.R., Guyre P.M., Pioli P.A. (2009). Functional expression of pattern recognition receptors in tissues of the human female reproductive tract. J. Reprod. Immunol..

[15] De Tomasi J.B., Opata M.M., Mowa C.N. (2019). Immunity in the Cervix: Interphase between Immune and Cervical Epithelial Cells. J. Immunol. Res..

[16] Farage M.A., Miller K.W., Gerberick G.F., Saito F., Ledger W., Witkin S. (2011). Innate immunity in the lower female mucosal tract. J. Steroids Horm. Sci..

[17] Quayle A.J. (2002). The innate and early immune response to pathogen challenge in the female genital tract and the pivotal role of epithelial cells. J. Reprod. Immunol..

[18] Fichorova R.N., Anderson D.J. (1999). Differential expression of immunobiological mediators by immortalized human cervical and vaginal epithelial cells. Biol. Reprod..

[19] Wira C.R., Grant-Tschudy K.S., Crane-Godreau M.A. (2005). Epithelial cells in the female reproductive tract: A central role as sentinels of immune protection. Am. J. Reprod. Immunol..

[20] Inki P. (1997). Expression of syndecan-1 in female reproductive tract tissues and cultured keratinocytes. Mol. Hum. Reprod..

[21] Gallo R.L., Nakatsuji T. (2011). Microbial Symbiosis with the Innate Immune Defense System of the Skin. J. Investig. Dermatol..

[22] Nelson A.M., Reddy S.K., Ratliff T.S., Hossain M.Z., Katseff A.S., Zhu A.S., Chang E., Resnik S.R., Page C., Kim D. (2015). dsRNA Released by Tissue Damage Activates TLR3 to Drive Skin Regeneration. Cell Stem Cell.

[23] Wang J.-N., Li M. (2020). The Immune Function of Keratinocytes in Anti-Pathogen Infection in the Skin. Int. J. Dermatol. Venereol..

[24] Chieosilapatham P., Kiatsurayanon C., Umehara Y., Trujillo-Paez J.V., Peng G., Yue H., Nguyen L.T.H., Niyonsaba F. (2021). Keratinocytes: Innate immune cells in atopic dermatitis. Clin. Exp. Immunol..

[25] Jiang Y., Tsoi L.C., Billi A.C., Ward N.L., Harms P.W., Zeng C., Maverakis E., Kahlenberg J.M., Gudjonsson J.E. (2020). Cytokinocytes: The diverse contribution of keratinocytes to immune responses in skin. JCI Insight.

[26] Bernfield M., Götte M., Park P.W., Reizes O., Fitzgerald M.L., Lincecum J., Zako M. (1999). Functions of cell surface heparan sulfate proteoglycans. Annu. Rev. Biochem..

[27] Szatmári T., Mundt F., Kumar-Singh A., Möbus L., Ötvös R., Hjerpe A., Dobra K. (2017). Molecular targets and signaling pathways regulated by nuclear translocation of syndecan-1. BMC Cell Biol..

[28] Rodriguez Garcia M., Patel M.V., Shen Z., Fahey J.V., Biswas N., Mestecky J., Wira C.R., Mestecky J., Strober W., Russell M.W., Kelsall B.L., Cheroutre H., Lambrecht B.N. (2015). Chapter 108—Mucosal Immunity in the Human Female Reproductive Tract. Mucosal Immunology.

[29] White H.D., Crassi K., Wira C. (1997). Cytolytic functional activities of NK cells and cytotoxic T lymphocytes (CTL) are coordinately regulated in the human female reproductive tract. Mucosal Solutions: Advances in Mucosal Immunology.

[30] Trifonova R.T., Lieberman J., van Baarle D. (2014). Distribution of Immune Cells in the Human Cervix and Implications for HIV Transmission. Am. J. Reprod. Immunol..

[31] Pudney J., Quayle A.J., Anderson D.J. (2005). Immunological Microenvironments in the Human Vagina and Cervix: Mediators of Cellular Immunity Are Concentrated in the Cervical Transformation Zone1. Biol. Reprod..

[32] Givan A.L., White H.D., Stern J.E., Colby E., Guyre P.M., Wira C.R., Gosselin E.J. (1997). Flow Cytometric Analysis of Leukocytes in the Human Female Reproductive Tract: Comparison of Fallopian Tube, Uterus, Cervix, and Vagina. Am. J. Reprod. Immunol..

[33] Kutteh W.H., Mestecky J., Wira C. (2005). Mucosal Immunity in the Human Female Reproductive Tract.

[34] Sakamoto Y., Moran P., Bulmer J.N., Searle R.F., Robson S.C. (2005). Macrophages and not granulocytes are involved in cervical ripening. J. Reprod. Immunol..

[35] Monin L., Whettlock E.M., Male V. (2020). Immune responses in the human female reproductive tract. Immunology.

[36] Wira C.R., Fahey J.V., Rodriguez-Garcia M., Shen Z., Patel M.V. (2014). Regulation of Mucosal Immunity in the Female Reproductive Tract: The Role of Sex Hormones in Immune Protection against Sexually Transmitted Pathogens. Am. J. Reprod. Immunol..

[37] Maucort-Boulch D., Franceschi S., Plummer M., Group IHPSS (2008). International correlation between human papillomavirus prevalence and cervical cancer incidence. Cancer Epidemiol. Biomark. Prev..

[38] Demarco M., Hyun N., Carter-Pokras O., Raine-Bennett T.R., Cheung L., Chen X., Hammer A., Campos N., Kinney W., Gage J.C. (2020). A study of type-specific HPV natural history and implications for contemporary cervical cancer screening programs. eClinicalMedicine.

[39] Zhang J., Jin S., Li X., Liu L., Xi L., Wang F., Zhang S. (2019). Human Papillomavirus Type 16 Disables the Increased Natural Killer Cells in Early Lesions of the Cervix. J. Immunol. Res..

[40] Bosch F.X., Burchell A.N., Schiffman M., Giuliano A.R., de Sanjose S., Bruni L., Tortolero-Luna G., Kjaer S.K., Muñoz N. (2008). Epidemiology and Natural History of Human Papillomavirus Infections and Type-Specific Implications in Cervical Neoplasia. Vaccine.

[41] Bruno M.T., Scalia G., Cassaro N., Boemi S. (2020). Multiple HPV 16 infection with two strains: A possible marker of neoplastic progression. BMC Cancer.

[42] Chen W., Zhang X., Molijn A., Jenkins D., Shi J.-F., Quint W., Schmidt J.E., Wang P., Liu Y.-L., Li L.-K. (2009). Human papillomavirus type-distribution in cervical cancer in China: The importance of HPV 16 and 18. Cancer Causes Control.

[43] Haghshenas M., Golini-Moghaddam T., Rafiei A., Emadeian O., Shykhpour A., Ashrafi G.H. (2013). Prevalence and type distribution of high-risk human papillomavirus in patients with cervical cancer: A population-based study. Infect. Agents Cancer.

[44] de Freitas A.C., de Oliveira T.H.A., Barros M.R., Venuti A. (2017). hrHPV E5 oncoprotein: Immune evasion and related immunotherapies. J. Exp. Clin. Cancer Res..

[45] Graham S.V. (2017). The human papillomavirus replication cycle, and its links to cancer progression: A comprehensive review. Clin. Sci..

[46] Basukala O., Banks L. (2021). The Not-So-Good, the Bad and the Ugly: HPV E5, E6 and E7 Oncoproteins in the Orchestration of Carcinogenesis. Viruses.

[47] Kumar V., Stewart J.H. (2023). Immunometabolic reprogramming, another cancer hallmark. Front. Immunol..

[48] Zhang R., Shen C., Zhao L., Wang J., McCrae M., Chen X., Lu F. (2016). Dysregulation of host cellular genes targeted by human papillomavirus (HPV) integration contributes to HPV-related cervical carcinogenesis. Int. J. Cancer.

[49] Moody C.A., Laimins L.A. (2010). Human papillomavirus oncoproteins: Pathways to transformation. Nat. Rev. Cancer.

[50] Stanley Margaret A. (2012). Epithelial Cell Responses to Infection with Human Papillomavirus. Clin. Microbiol. Rev..

[51] Fritz J.H., Le Bourhis L., Magalhaes J.G., Philpott D.J. (2008). Innate immune recognition at the epithelial barrier drives adaptive immunity: APCs take the back seat. Trends Immunol..

[52] Tindle R.W. (2002). Immune evasion in human papillomavirus-associated cervical cancer. Nat. Rev. Cancer.

[53] Uhlorn B.L., Jackson R., Li S., Bratton S.M., Van Doorslaer K., Campos S.K. (2020). Vesicular trafficking permits evasion of cGAS/STING surveillance during initial human papillomavirus infection. PLoS Pathog..

[54] Berger K.L., Barriga F., Lace M.J., Turek L.P., Zamba G.J., Domann F.E., Lee J.H., Klingelhutz A.J. (2006). Cervical keratinocytes containing stably replicating extrachromosomal HPV-16 are refractory to transformation by oncogenic H-Ras. Virology.

[55] Pett M.R., Herdman M.T., Palmer R.D., Yeo G.S.H., Shivji M.K., Stanley M.A., Coleman N. (2006). Selection of cervical keratinocytes containing integrated HPV16 associates with episome loss and an endogenous antiviral response. Proc. Natl. Acad. Sci. USA.

[56] De Leo A., Calderon A., Lieberman P.M. (2020). Control of Viral Latency by Episome Maintenance Proteins. Trends Microbiol..

[57] Nees M., Geoghegan J.M., Hyman T., Frank S., Miller L., Woodworth C.D. (2001). Papillomavirus type 16 oncogenes downregulate expression of interferon-responsive genes and upregulate proliferation-associated and NF-kappaB-responsive genes in cervical keratinocytes. J. Virol..

[58] Lau L., Gray E.E., Brunette R.L., Stetson D.B. (2015). DNA tumor virus oncogenes antagonize the cGAS-STING DNA-sensing pathway. Science.

[59] Lou M., Huang D., Zhou Z., Shi X., Wu M., Rui Y., Su J., Zheng W., Yu X.-F. (2023). DNA virus oncoprotein HPV18 E7 selectively antagonizes cGAS-STING-triggered innate immune activation. J. Med. Virol..

[60] Xiao D., Huang W., Ou M., Guo C., Ye X., Liu Y., Wang M., Zhang B., Zhang N., Huang S. (2016). Interaction between susceptibility loci in cGAS-STING pathway, MHC gene and HPV infection on the risk of cervical precancerous lesions in Chinese population. Oncotarget.

[61] Berti F.C.B., Pereira A.P.L., Cebinelli G.C.M., Trugilo K.P., Brajão de Oliveira K. (2017). The role of interleukin 10 in human papilloma virus infection and progression to cervical carcinoma. Cytokine Growth Factor. Rev..

[62] Nees M., Geoghegan J.M., Munson P., Prabhu V., Liu Y., Androphy E., Woodworth C.D. (2000). Human papillomavirus type 16 E6 and E7 proteins inhibit differentiation-dependent expression of transforming growth factor-beta2 in cervical keratinocytes. Cancer Res..

[63] Iglesias M., Yen K., Gaiotti D., Hildesheim A., Stoler M.H., Woodworth C.D. (1998). Human papillomavirus type 16 E7 protein sensitizes cervical keratinocytes to apoptosis and release of interleukin-1alpha. Oncogene.

[64] Shimada M., Yamashita A., Saito M., Ichino M., Kinjo T., Mizuki N., Klinman D.M., Okuda K. (2020). The human papillomavirus E6 protein targets apoptosis-inducing factor (AIF) for degradation. Sci. Rep..

[65] Guo L., Hua K.A.-O. (2020). Cervical Cancer: Emerging Immune Landscape and Treatment. OncoTargets Ther..

[66] Ghosh M., Saha S., Li J., Montrose D.C., Martinez L.A. (2023). p53 engages the cGAS/STING cytosolic DNA sensing pathway for tumor suppression. Mol. Cell.

[67] Zhang S., Wang H., Liu J., Tao T., Zeng Z., Wang M. (2022). RGS1 and related genes as potential targets for immunotherapy in cervical cancer: Computational biology and experimental validation. J. Transl. Med..

[68] Agenès F., Bosco N., Mascarell L., Fritah S., Ceredig R. (2005). Differential expression of regulator of G-protein signalling transcripts and in vivo migration of CD4+ naïve and regulatory T cells. Immunology.

[69] Shi G.X., Harrison K., Han S.B., Moratz C., Kehrl J.H. (2004). Toll-like receptor signaling alters the expression of regulator of G protein signaling proteins in dendritic cells: Implications for G protein-coupled receptor signaling. J. Immunol..

[70] Bai Y., Hu M., Chen Z., Wei J., Du H. (2021). Single-Cell Transcriptome Analysis Reveals RGS1 as a New Marker and Promoting Factor for T-Cell Exhaustion in Multiple Cancers. Front. Immunol..

[71] Kang Y., Huang J., Liu Y., Zhang N., Cheng Q., Zhang Y. (2021). Integrated Analysis of Immune Infiltration Features for Cervical Carcinoma and Their Associated Immunotherapeutic Responses. Front. Cell Dev. Biol..

[72] Yamato K., Yamada T., Kizaki M., Ui-Tei K., Natori Y., Fujino M., Nishihara T., Ikeda Y., Nasu Y., Saigo K. (2008). New highly potent and specific E6 and E7 siRNAs for treatment of HPV16 positive cervical cancer. Cancer Gene Ther..

[73] Bergot A.S., Ford N., Leggatt G.R., Wells J.W., Frazer I.H., Grimbaldeston M.A. (2014). HPV16-E7 expression in squamous epithelium creates a local immune suppressive environment via CCL2- and CCL5- mediated recruitment of mast cells. PLoS Pathog..

[74] Pal A., Kundu R. (2020). Human Papillomavirus E6 and E7: The Cervical Cancer Hallmarks and Targets for Therapy. Front. Microbiol..

[75] Taghizadeh E., Jahangiri S., Rostami D., Taheri F., Renani P.G., Taghizadeh H., Gheibi Hayat S.M. (2019). Roles of E6 and E7 Human Papillomavirus Proteins in Molecular Pathogenesis of Cervical Cancer. Curr. Protein Pept. Sci..

[76] Romani N., Brunner P.M., Stingl G. (2012). Changing Views of the Role of Langerhans Cells. J. Investig. Dermatol..

[77] Doebel T., Voisin B., Nagao K. (2017). Langerhans Cells—The Macrophage in Dendritic Cell Clothing. Trends Immunol..

[78] Merad M., Ginhoux F., Collin M. (2008). Origin, homeostasis and function of Langerhans cells and other langerin-expressing dendritic cells. Nat. Rev. Immunol..

[79] Woodham A.W., Raff A.B., Raff L.M., Da Silva D.M., Yan L., Skeate J.G., Wong M.K., Lin Y.G., Kast W.M. (2014). Inhibition of Langerhans cell maturation by human papillomavirus type 16: A novel role for the annexin A2 heterotetramer in immune suppression. J. Immunol..

[80] Da Silva D.M., Woodham A.W., Skeate J.G., Rijkee L.K., Taylor J.R., Brand H.E., Muderspach L.I., Roman L.D., Yessaian A.A., Pham H.Q. (2015). Langerhans cells from women with cervical precancerous lesions become functionally responsive against human papillomavirus after activation with stabilized Poly-I:C. Clin. Immunol..

[81] Fahey L.M., Raff A.B., Da Silva D.M., Kast W.M. (2009). Reversal of human papillomavirus-specific T cell immune suppression through TLR agonist treatment of Langerhans cells exposed to human papillomavirus type 16. J. Immunol..

[82] Fausch S.C., Da Silva D.M., Kast W.M. (2005). Heterologous papillomavirus virus-like particles and human papillomavirus virus-like particle immune complexes activate human Langerhans cells. Vaccine.

[83] Vignali D.A.A., Kuchroo V.K. (2012). IL-12 family cytokines: Immunological playmakers. Nat. Immunol..

[84] Gee K., Guzzo C., Che Mat N.F., Ma W., Kumar A. (2009). The IL-12 family of cytokines in infection, inflammation and autoimmune disorders. Inflamm. Allergy Drug. Targets.

[85] García-Piñeres A.J., Hildesheim A., Trivett M., Williams M., Wu L., Kewalramani V.N., Pinto L.A. (2006). Role of DC-SIGN in the activation of dendritic cells by HPV-16 L1 virus-like particle vaccine. Eur. J. Immunol..

[86] Woodham A.W., Yan L., Skeate J.G., van der Veen D., Brand H.H., Wong M.K., Da Silva D.M., Kast W.M. (2016). T cell ignorance is bliss: T cells are not tolerized by Langerhans cells presenting human papillomavirus antigens in the absence of costimulation. Papillomavirus Res..

[87] Reyna-Hernández M.A., Alarcón-Romero L.D.C., Ortiz-Ortiz J., Illades-Aguiar B., Jiménez-López M.A., Ocampo-Bárcenas A., Morrugares-Ixtepan M.O., Torres-Rojas F.I. (2022). GLUT1, LDHA, and MCT4 Expression Is Deregulated in Cervical Cancer and Precursor Lesions. J. Histochem. Cytochem..

[88] Bedoya A.M., Tate D.J., Baena A., Córdoba C.M., Borrero M., Pareja R., Rojas F., Patterson J.R., Herrero R., Zea A.H. (2014). Immunosuppression in cervical cancer with special reference to arginase activity. Gynecol. Oncol..

[89] Caldwell R.W., Rodriguez P.C., Toque H.A., Narayanan S.P., Caldwell R.B. (2018). Arginase: A Multifaceted Enzyme Important in Health and Disease. Physiol. Rev..

[90] Casero R.A., Murray Stewart T., Pegg A.E. (2018). Polyamine metabolism and cancer: Treatments, challenges and opportunities. Nat. Rev. Cancer.

[91] Gerner E.W., Meyskens F.L. (2004). Polyamines and cancer: Old molecules, new understanding. Nat. Rev. Cancer.

[92] Souid M., Ghedira R., Souissi S., Bouzgarrou N., Gabbouj S., Shini-Hadhri S., Rhim M.-S., Boukadida A., Toumi D., Faleh R. (2022). Arginase is involved in cervical lesions progression and severity. Immunobiology.

[93] Venancio P.A., Consolaro M.E.L., Derchain S.F., Boccardo E., Villa L.L., Maria-Engler S.S., Campa A., Discacciati M.G. (2019). Indoleamine 2,3-dioxygenase and tryptophan 2,3-dioxygenase expression in HPV infection, SILs, and cervical cancer. Cancer Cytopathol..

[94] Hascitha J., Priya R., Jayavelu S., Dhandapani H., Selvaluxmy G., Sunder Singh S., Rajkumar T. (2016). Analysis of Kynurenine/Tryptophan ratio and expression of IDO1 and 2 mRNA in tumour tissue of cervical cancer patients. Clin. Biochem..

[95] Ferns D.M., Kema I.P., Buist M.R., Nijman H.W., Kenter G.G., Jordanova E.S. (2015). Indoleamine-2,3-dioxygenase (IDO) metabolic activity is detrimental for cervical cancer patient survival. OncoImmunology.

[96] Yang S.L., Tan H.X., Niu T.T., Liu Y.K., Gu C.J., Li D.J., Li M.Q., Wang H.Y. (2021). The IFN-γ-IDO1-kynureine pathway-induced autophagy in cervical cancer cell promotes phagocytosis of macrophage. Int. J. Biol. Sci..

[97] Hu Y., Liu Z., Tang H. (2022). Tryptophan 2,3-dioxygenase may be a potential prognostic biomarker and immunotherapy target in cancer: A meta-analysis and bioinformatics analysis. Front. Oncol..

[98] Wang J., Mijiti Y., Chen Y., Liu Z. (2021). Aryl hydrocarbon receptor is a prognostic biomarker and is correlated with immune responses in cervical cancer. Bioengineered.

[99] Opitz C.A., Litzenburger U.M., Sahm F., Ott M., Tritschler I., Trump S., Schumacher T., Jestaedt L., Schrenk D., Weller M. (2011). An endogenous tumour-promoting ligand of the human aryl hydrocarbon receptor. Nature.

[100] Feng S., Cao Z., Wang X. (2013). Role of aryl hydrocarbon receptor in cancer. Biochim. Biophys. Acta (BBA)-Rev. Cancer.

[101] Gasiewicz T.A., Henry E.C., Collins L.L. (2008). Expression and Activity of Aryl Hydrocarbon Receptors in Development and Cancer. Crit. Rev. Eukaryot. Gene Expr..

[102] Marvin D.L., Spaans V.M., de Kroon C.D., Slieker R.C., Khelil M., Ten Dijke P., Ritsma L., Jordanova E.S. (2022). Low Transforming Growth Factor-β Pathway Activity in Cervical Adenocarcinomas. Front. Oncol..

[103] Labani-Motlagh A., Ashja-Mahdavi M., Loskog A. (2020). The Tumor Microenvironment: A Milieu Hindering and Obstructing Antitumor Immune Responses. Front. Immunol..

[104] Falcomatà C., Bärthel S., Schneider G., Rad R., Schmidt-Supprian M., Saur D. (2023). Context-Specific Determinants of the Immunosuppressive Tumor Microenvironment in Pancreatic Cancer. Cancer Discov..

[105] Wang Q., Shao X., Zhang Y., Zhu M., Wang F.X.C., Mu J., Li J., Yao H., Chen K. (2023). Role of tumor microenvironment in cancer progression and therapeutic strategy. Cancer Med..

[106] Piersma S.J. (2011). Immunosuppressive tumor microenvironment in cervical cancer patients. Cancer Microenviron..

[107] Yang X., Zhang W., Zhu W. (2023). Profiling of immune responses by lactate modulation in cervical cancer reveals key features driving clinical outcome. Heliyon.

[108] Zhao M., Li Y., Wei X., Zhang Q., Jia H., Quan S., Cao D., Wang L., Yang T., Zhao J. (2017). Negative immune factors might predominate local tumor immune status and promote carcinogenesis in cervical carcinoma. Virol. J..

[109] Xu J., Huang Z., Wang Y., Xiang Z., Xiong B. (2022). Identification of Novel Tumor Microenvironment Regulating Factor That Facilitates Tumor Immune Infiltration in Cervical Cancer. Front. Oncol..

[110] Miyata Y., Ogo E., Abe T., Hirata H., Tsuda N., Ushijima K., Kawahara A., Akiba J., Obara H., Kakuma T. (2023). Dynamics in the expression of programmed death ligand 1 and cluster of differentiation 163 in the tumor microenvironment of uterine cervical cancer: A single-center retrospective study. Radiat. Oncol..

[111] Pu Y., Ji Q. (2022). Tumor-Associated Macrophages Regulate PD-1/PD-L1 Immunosuppression. Front. Immunol..

[112] Huang W., Liu J., Xu K., Chen H., Bian C. (2022). PD-1/PD-L1 inhibitors for advanced or metastatic cervical cancer: From bench to bed. Front. Oncol..

[113] Mezache L., Paniccia B., Nyinawabera A., Nuovo G.J. (2015). Enhanced expression of PD L1 in cervical intraepithelial neoplasia and cervical cancers. Mod. Pathol..

[114] Liu C., Lu J., Tian H., Du W., Zhao L., Feng J., Yuan D., Li Z. (2017). Increased expression of PD-L1 by the human papillomavirus 16 E7 oncoprotein inhibits anticancer immunity. Mol. Med. Rep..

[115] Yang W., Lu Y.P., Yang Y.Z., Kang J.R., Jin Y.D., Wang H.W. (2017). Expressions of programmed death (PD)-1 and PD-1 ligand (PD-L1) in cervical intraepithelial neoplasia and cervical squamous cell carcinomas are of prognostic value and associated with human papillomavirus status. J. Obs. Gynaecol. Res..

[116] Meng Y., Liang H., Hu J., Liu S., Hao X., Wong M.S.K., Li X., Hu L. (2018). PD-L1 Expression Correlates with Tumor Infiltrating Lymphocytes and Response to Neoadjuvant Chemotherapy in Cervical Cancer. J. Cancer.

[117] Yang-Chun F., Zhen-Zhen C., Yan-Chun H., Xiu-Min M. (2017). Association between PD-L1 and HPV status and the prognostic value for HPV treatment in premalignant cervical lesion patients. Medicine.

[118] Yao S., Zhao L., Chen S., Wang H., Gao Y., Shao N.-Y., Dai M., Cai H. (2023). Cervical cancer immune infiltration microenvironment identification, construction of immune scores, assisting patient prognosis and immunotherapy. Front. Immunol..

[119] Walker L.S.K. (2013). Treg and CTLA-4: Two intertwining pathways to immune tolerance. J. Autoimmun..

[120] Imazeki H., Ogiwara Y., Kawamura M., Boku N., Kudo-Saito C. (2021). CD11b(+)CTLA4(+) myeloid cells are a key driver of tumor evasion in colorectal cancer. J. Immunother. Cancer.

[121] Li H., van der Leun A.M., Yofe I., Lubling Y., Gelbard-Solodkin D., van Akkooi A.C.J., van den Braber M., Rozeman E.A., Haanen J., Blank C.U. (2019). Dysfunctional CD8 T Cells Form a Proliferative, Dynamically Regulated Compartment within Human Melanoma. Cell.

[122] Yang X., Chen Y., Li M., Zhu W. (2022). ERBB3 methylation and immune infiltration in tumor microenvironment of cervical cancer. Sci. Rep..

[123] Audirac-Chalifour A., Torres-Poveda K., Bahena-Román M., Téllez-Sosa J., Martínez-Barnetche J., Cortina-Ceballos B., López-Estrada G., Delgado-Romero K., Burguete-García A.I., Cantú D. (2016). Cervical Microbiome and Cytokine Profile at Various Stages of Cervical Cancer: A Pilot Study. PLoS ONE.

[124] Kyrgiou M., Moscicki A.-B. (2022). Vaginal microbiome and cervical cancer. Semin. Cancer Biol..

[125] Vaccarella S., Lortet-Tieulent J., Plummer M., Franceschi S., Bray F. (2013). Worldwide trends in cervical cancer incidence: Impact of screening against changes in disease risk factors. Eur. J. Cancer.

[126] Castanheira C.P., Sallas M.L., Nunes R.A.L., Lorenzi N.P.C., Termini L. (2021). Microbiome and Cervical Cancer. Pathobiology.

[127] Tang Y., Zhang A.X.J., Chen G., Wu Y., Gu W. (2021). Prognostic and therapeutic TILs of cervical cancer—Current advances and future perspectives. Mol. Ther.-Oncolytics.

[128] Binnewies M., Roberts E.W., Kersten K., Chan V., Fearon D.F., Merad M., Coussens L.M., Gabrilovich D.I., Ostrand-Rosenberg S., Hedrick C.C. (2018). Understanding the tumor immune microenvironment (TIME) for effective therapy. Nat. Med..

[129] Frankel T., Lanfranca M.P., Zou W. (2017). The Role of Tumor Microenvironment in Cancer Immunotherapy. Adv. Exp. Med. Biol..

[130] Ostuni R., Kratochvill F., Murray P.J., Natoli G. (2015). Macrophages and cancer: From mechanisms to therapeutic implications. Trends Immunol..

[131] Ma R.-Y., Black A., Qian B.-Z. (2022). Macrophage diversity in cancer revisited in the era of single-cell omics. Trends Immunol..

[132] Kumar V., Khalid Hussain B. (2019). Macrophages: The Potent Immunoregulatory Innate Immune Cells. Macrophage Activation.

[133] Liu J., Geng X., Hou J., Wu G. (2021). New insights into M1/M2 macrophages: Key modulators in cancer progression. Cancer Cell Int..

[134] Li C., Hua K. (2022). Dissecting the Single-Cell Transcriptome Network of Immune Environment Underlying Cervical Premalignant Lesion, Cervical Cancer and Metastatic Lymph Nodes. Front. Immunol..

[135] Mantovani A., Allavena P., Marchesi F., Garlanda C. (2022). Macrophages as tools and targets in cancer therapy. Nat. Rev. Drug. Discov..

[136] Nuñez S.Y., Ziblat A., Secchiari F., Torres N.I., Sierra J.M., Raffo Iraolagoitia X.L., Araya R.E., Domaica C.I., Fuertes M.B., Zwirner N.W. (2018). Human M2 Macrophages Limit NK Cell Effector Functions through Secretion of TGF-β and Engagement of CD85j. J. Immunol..

[137] Guo F., Feng Y.C., Zhao G., Zhang R., Cheng Z.Z., Kong W.N., Wu H.L., Xu B., Lv X., Ma X.M. (2020). Tumor-Associated CD163(+) M2 Macrophage Infiltration is Highly Associated with PD-L1 Expression in Cervical Cancer. Cancer Manag. Res..

[138] Chen X.-J., Wu S., Yan R.-M., Fan L.-S., Yu L., Zhang Y.-M., Wei W.-F., Zhou C.-F., Wu X.-G., Zhong M. (2019). The role of the hypoxia-Nrp-1 axis in the activation of M2-like tumor-associated macrophages in the tumor microenvironment of cervical cancer. Mol. Carcinog..

[139] Ren J., Li L., Yu B., Xu E., Sun N., Li X., Xing Z., Han X., Cui Y., Wang X. (2022). Extracellular vesicles mediated proinflammatory macrophage phenotype induced by radiotherapy in cervical cancer. BMC Cancer.

[140] Krneta T., Gillgrass A., Poznanski S., Chew M., Lee A.J., Kolb M., Ashkar A.A. (2017). M2-polarized and tumor-associated macrophages alter NK cell phenotype and function in a contact-dependent manner. J. Leukoc. Biol..

[141] Wang S., Yang Y., Ma P., Zha Y., Zhang J., Lei A., Li N. (2022). CAR-macrophage: An extensive immune enhancer to fight cancer. eBioMedicine.

[142] Klichinsky M., Ruella M., Shestova O., Lu X.M., Best A., Zeeman M., Schmierer M., Gabrusiewicz K., Anderson N.R., Petty N.E. (2020). Human chimeric antigen receptor macrophages for cancer immunotherapy. Nat. Biotechnol..

[143] Shaul M.E., Fridlender Z.G. (2019). Tumour-associated neutrophils in patients with cancer. Nat. Rev. Clin. Oncol..

[144] Hedrick C.C., Malanchi I. (2022). Neutrophils in cancer: Heterogeneous and multifaceted. Nat. Rev. Immunol..

[145] Jaillon S., Ponzetta A., Di Mitri D., Santoni A., Bonecchi R., Mantovani A. (2020). Neutrophil diversity and plasticity in tumour progression and therapy. Nat. Rev. Cancer.

[146] Coffelt S.B., Wellenstein M.D., de Visser K.E. (2016). Neutrophils in cancer: Neutral no more. Nat. Rev. Cancer.

[147] Nomelini R.S., Mota S.D.S., Murta E.F.C. (2022). Absolute band neutrophils count is a predictor of overall survival in advanced uterine cervical cancer. Arch. Gynecol. Obstet..

[148] Carus A., Ladekarl M., Hager H., Nedergaard B.S., Donskov F. (2013). Tumour-associated CD66b+ neutrophil count is an independent prognostic factor for recurrence in localised cervical cancer. Br. J. Cancer.

[149] Yan B., Dai X., Ma Q., Wu X. (2021). Stromal Neutrophil Extracellular Trap Density Is an Independent Prognostic Factor for Cervical Cancer Recurrence. Front. Oncol..

[150] Punt S., Fleuren G.J., Kritikou E., Lubberts E., Trimbos J.B., Jordanova E.S., Gorter A. (2015). Angels and demons: Th17 cells represent a beneficial response, while neutrophil IL-17 is associated with poor prognosis in squamous cervical cancer. Oncoimmunology.

[151] Ittiamornlert P., Ruengkhachorn I. (2019). Neutrophil-lymphocyte ratio as a predictor of oncologic outcomes in stage IVB, persistent, or recurrent cervical cancer patients treated by chemotherapy. BMC Cancer.

[152] Calo C.A., Barrington D.A., Brown M., Gonzalez L., Baek J., Huffman A., Benedict J., Backes F., Chambers L., Cohn D. (2022). High pre-treatment neutrophil-to-lymphocyte ratio as a prognostic marker for worse survival in patients with recurrent/metastatic cervical cancer treated with immune checkpoint inhibitors. Gynecol. Oncol. Rep..

[153] Gruijs M., Sewnath C.A.N., van Egmond M. (2021). Therapeutic exploitation of neutrophils to fight cancer. Semin. Immunol..

[154] Carnevale S., Ghasemi S., Rigatelli A., Jaillon S. (2020). The complexity of neutrophils in health and disease: Focus on cancer. Semin. Immunol..

[155] Geh D., Leslie J., Rumney R., Reeves H.L., Bird T.G., Mann D.A. (2022). Neutrophils as potential therapeutic targets in hepatocellular carcinoma. Nat. Rev. Gastroenterol. Hepatol..

[156] Németh T., Sperandio M., Mócsai A. (2020). Neutrophils as emerging therapeutic targets. Nat. Rev. Drug Discov..

[157] Groth C., Hu X., Weber R., Fleming V., Altevogt P., Utikal J., Umansky V. (2019). Immunosuppression mediated by myeloid-derived suppressor cells (MDSCs) during tumour progression. Br. J. Cancer.

[158] Kumar V., Patel S., Tcyganov E., Gabrilovich D.I. (2016). The Nature of Myeloid-Derived Suppressor Cells in the Tumor Microenvironment. Trends Immunol..

[159] Raskov H., Orhan A., Gaggar S., Gögenur I. (2022). Neutrophils and polymorphonuclear myeloid-derived suppressor cells: An emerging battleground in cancer therapy. Oncogenesis.

[160] Wesolowski R., Markowitz J., Carson W.E. (2013). Myeloid derived suppressor cells—A new therapeutic target in the treatment of cancer. J. Immunotherapy Cancer.

[161] Kawano M., Mabuchi S., Matsumoto Y., Sasano T., Takahashi R., Kuroda H., Kozasa K., Hashimoto K., Isobe A., Sawada K. (2015). The significance of G-CSF expression and myeloid-derived suppressor cells in the chemoresistance of uterine cervical cancer. Sci. Rep..

[162] Sasano T., Mabuchi S., Kozasa K., Kuroda H., Kawano M., Takahashi R., Komura N., Yokoi E., Matsumoto Y., Hashimoto K. (2018). The Highly Metastatic Nature of Uterine Cervical/Endometrial Cancer Displaying Tumor-Related Leukocytosis: Clinical and Preclinical Investigations. Clin. Cancer Res..

[163] Cho Y., Kim K.H., Yoon H.I., Kim G.E., Kim Y.B. (2016). Tumor-related leukocytosis is associated with poor radiation response and clinical outcome in uterine cervical cancer patients. Ann. Oncol..

[164] Nagaraj S., Youn J.I., Gabrilovich D.I. (2013). Reciprocal relationship between myeloid-derived suppressor cells and T cells. J. Immunol..

[165] Lu Z., Zhu M., Marley J.L., Bi K., Wang K., Zhai M., Hu H., Guo P., Li C., Xu Y. (2021). The combined action of monocytic myeloid-derived suppressor cells and mucosal-associated invariant T cells promotes the progression of cervical cancer. Int. J. Cancer.

[166] Liang Y., Wang W., Zhu X., Yu M., Zhou C. (2022). Inhibition of myeloid-derived suppressive cell function with all-trans retinoic acid enhanced anti-PD-L1 efficacy in cervical cancer. Sci. Rep..

[167] Kumar V., Ahmad A. (2018). Role of MAIT cells in the immunopathogenesis of inflammatory diseases: New players in old game. Int. Rev. Immunol..

[168] Ussher J.E., Willberg C.B., Klenerman P. (2018). MAIT cells and viruses. Immunol. Cell Biol..

[169] Rouxel O., Lehuen A. (2018). Mucosal-associated invariant T cells in autoimmune and immune-mediated diseases. Immunol. Cell Biol..

[170] Hinks T.S.C., van Wilgenburg B., Wang H., Loh L., Koutsakos M., Kedzierska K., Corbett A.J., Chen Z. (2020). Study of MAIT Cell Activation in Viral Infections In Vivo. Methods Mol. Biol..

[171] Zumwalde N.A., Gumperz J.E. (2020). Mucosal-Associated Invariant T Cells in Tumors of Epithelial Origin. Adv. Exp. Med. Biol..

[172] Berzins S.P., Wallace M.E., Kannourakis G., Kelly J. (2020). A Role for MAIT Cells in Colorectal Cancer. Front. Immunol..

[173] Shaler C.R., Tun-Abraham M.E., Skaro A.I., Khazaie K., Corbett A.J., Mele T., Hernandez-Alejandro R., Haeryfar S.M.M. (2017). Mucosa-associated invariant T cells infiltrate hepatic metastases in patients with colorectal carcinoma but are rendered dysfunctional within and adjacent to tumor microenvironment. Cancer Immunol. Immunother..

[174] Haeryfar S.M.M., Shaler C.R., Rudak P.T. (2018). Mucosa-associated invariant T cells in malignancies: A faithful friend or formidable foe?. Cancer Immunol. Immunother..

[175] Melo A.M., O’Brien A.M., Phelan J.J., Kennedy S.A., Wood N.A.W., Veerapen N., Besra G.S., Clarke N.E., Foley E.K., Ravi A. (2019). Mucosal-Associated Invariant T Cells Display Diminished Effector Capacity in Oesophageal Adenocarcinoma. Front. Immunol..

[176] O’Neill C., Cassidy F.C., O’Shea D., Hogan A.E. (2021). Mucosal Associated Invariant T Cells in Cancer-Friend or Foe?. Cancers.

[177] Li Y.-R., Zhou K., Wilson M., Kramer A., Zhu Y., Dawson N., Yang L. (2023). Mucosal-associated invariant T cells for cancer immunotherapy. Mol. Ther..

[178] Huang W.-C., Hsiao Y.-C., Wu C.-C., Hsu Y.-T., Chang C.-L. (2019). Less circulating mucosal-associated invariant T cells in patients with cervical cancer. Taiwan. J. Obstet. Gynecol..

[179] Yan J., Allen S., McDonald E., Das I., Mak J.Y.W., Liu L., Fairlie D.P., Meehan B.S., Chen Z., Corbett A.J. (2020). MAIT Cells Promote Tumor Initiation, Growth, and Metastases via Tumor MR1. Cancer Discov..

[180] Ruf B., Catania V.V., Wabitsch S., Ma C., Diggs L.P., Zhang Q., Heinrich B., Subramanyam V., Cui L.L., Pouzolles M. (2021). Activating Mucosal-Associated Invariant T Cells Induces a Broad Antitumor Response. Cancer Immunol. Res..

[181] Kolkhir P., Elieh-Ali-Komi D., Metz M., Siebenhaar F., Maurer M. (2022). Understanding human mast cells: Lesson from therapies for allergic and non-allergic diseases. Nat. Rev. Immunol..

[182] Kumar V., Sharma A. (2010). Mast cells: Emerging sentinel innate immune cells with diverse role in immunity. Mol. Immunol..

[183] Abraham S.N., St John A.L. (2010). Mast cell-orchestrated immunity to pathogens. Nat. Rev. Immunol..

[184] Galli S.J., Nakae S., Tsai M. (2005). Mast cells in the development of adaptive immune responses. Nat. Immunol..

[185] Aponte-López A., Muñoz-Cruz S. (2020). Mast Cells in the Tumor Microenvironment. Adv. Exp. Med. Biol..

[186] Komi D.E.A., Redegeld F.A. (2020). Role of Mast Cells in Shaping the Tumor Microenvironment. Clin. Rev. Allergy Immunol..

[187] Cabanillas-Saez A., Schalper J.A., Nicovani S.M., Rudolph M.I. (2002). Characterization of mast cells according to their content of tryptase and chymase in normal and neoplastic human uterine cervix. Int. J. Gynecol. Cancer.

[188] Naik R., Pai M.R., Poornima Baliga B., Nayak K.S., Shankarnarayana, Dighe P. (2004). Mast cell profile in uterine cervix. Indian J. Pathol. Microbiol..

[189] Kalyani R., Rajeshwari G. (2016). Significance of mast cells in non-neoplastic and neoplastic lesions of uterine cervix. Biomed. Res. Ther..

[190] Guo F., Kong W.N., Li D.W., Zhao G., Wu H.L., Anwar M., Shang X.Q., Sun Q.N., Ma C.L., Ma X.M. (2022). Low Tumor Infiltrating Mast Cell Density Reveals Prognostic Benefit in Cervical Carcinoma. Technol. Cancer Res. Treat..

[191] Benítez-Bribiesca L., Wong A., Utrera D., Castellanos E. (2001). The role of mast cell tryptase in neoangiogenesis of premalignant and malignant lesions of the uterine cervix. J. Histochem. Cytochem..

[192] Somasundaram R., Connelly T., Choi R., Choi H., Samarkina A., Li L., Gregorio E., Chen Y., Thakur R., Abdel-Mohsen M. (2021). Tumor-infiltrating mast cells are associated with resistance to anti-PD-1 therapy. Nat. Commun..

[193] Li J., Peng G., Zhu K., Jie X., Xu Y., Rao X., Xu Y., Chen Y., Xing B., Wu G. (2023). PD-1+ mast cell enhanced by PD-1 blocking therapy associated with resistance to immunotherapy. Cancer Immunol. Immunother..

[194] Shamri R., Xenakis J.J., Spencer L.A. (2011). Eosinophils in innate immunity: An evolving story. Cell Tissue Res..

[195] Travers J., Rothenberg M.E. (2015). Eosinophils in mucosal immune responses. Mucosal Immunol..

[196] Rothenberg M.E., Hogan S.P. (2006). The eosinophil. Annu. Rev. Immunol..

[197] Kita H. (2011). Eosinophils: Multifaceted biological properties and roles in health and disease. Immunol. Rev..

[198] Wen T., Rothenberg M.E. (2016). The Regulatory Function of Eosinophils. Microbiol. Spectr..

[199] Ravin K.A., Loy M. (2016). The Eosinophil in Infection. Clin. Rev. Allergy Immunol..

[200] Blanchard C., Rothenberg M.E. (2009). Chapter 3 Biology of the Eosinophil. Advances in Immunology.

[201] Kurose N., Mizuguchi S., Ohkanemasa Y., Yamashita M., Nakano M., Guo X., Aikawa A., Nakada S., Sasagawa T., Yamada S. (2019). Adenosquamous carcinoma of the uterine cervix displaying tumor-associated tissue eosinophilia. SAGE Open Med. Case Rep..

[202] van Driel W.J., Hogendoorn P.C.W., Jansen F.-W., Zwinderman A.H., Trimbos J.B., Fleuren G.J. (1996). Tumor-associated eosinophilic infiltrate of cervical cancer is indicative for a less effective immune response. Hum. Pathol..

[203] Höckel M., Schlenger K., Aral B., Mitze M., Schäffer U., Vaupel P. (1996). Association between Tumor Hypoxia and Malignant Progression in Advanced Cancer of the Uterine Cervix1. Cancer Res..

[204] Xie F., Meng Y.-H., Liu L.-B., Chang K.-K., Li H., Li M.-Q., Li D.-J. (2013). Cervical Carcinoma Cells Stimulate the Angiogenesis through TSLP Promoting Growth and Activation of Vascular Endothelial Cells. Am. J. Reprod. Immunol..

[205] Grisaru-Tal S., Itan M., Klion A.D., Munitz A. (2020). A new dawn for eosinophils in the tumour microenvironment. Nat. Rev. Cancer.

[206] Grisaru-Tal S., Rothenberg M.E., Munitz A. (2022). Eosinophil–lymphocyte interactions in the tumor microenvironment and cancer immunotherapy. Nat. Immunol..

[207] Steinman R.M., Banchereau J. (2007). Taking dendritic cells into medicine. Nature.

[208] Sakref C., Bendriss-Vermare N., Valladeau-Guilemond J. (2023). Phenotypes and Functions of Human Dendritic Cell Subsets in the Tumor Microenvironment. Methods Mol. Biol..

[209] Carenza C., Calcaterra F., Oriolo F., Di Vito C., Ubezio M., Della Porta M.G., Mavilio D., Della Bella S. (2019). Costimulatory Molecules and Immune Checkpoints Are Differentially Expressed on Different Subsets of Dendritic Cells. Front. Immunol..

[210] Spits H., Bernink J.H., Lanier L. (2016). NK cells and type 1 innate lymphoid cells: Partners in host defense. Nat. Immunol..

[211] Kumar V., Bauer C., Stewart J.H. (2022). Chasing Uterine Cancer with NK Cell-Based Immunotherapies. Future Pharmacol..

[212] Wolf N.K., Kissiov D.U., Raulet D.H. (2023). Roles of natural killer cells in immunity to cancer, and applications to immunotherapy. Nat. Rev. Immunol..

[213] Tu Y., Pan M., Song S., Hua J., Liu R., Li L. (2019). CD3+CD56+ natural killer T cell infiltration is increased in cervical cancer and negatively correlated with tumour progression. Biotechnol. Biotechnol. Equip..

[214] Céspedes M.A., Rodríguez J.A., Medina M., Bravo M., Cómbita A.L. (2012). Analysis of NK Cells in Peripheral Blood and Tumor Infiltrating Lymphocytes in Cervical Cancer Patients. Rev. Colomb. Cancerol..

[215] Gooden M., Lampen M., Jordanova E.S., Leffers N., Trimbos J.B., van der Burg S.H., Nijman H., van Hall T. (2011). HLA-E expression by gynecological cancers restrains tumor-infiltrating CD8(+) T lymphocytes. Proc. Natl. Acad. Sci. USA.

[216] Spaans V.M., Peters A.A.W., Fleuren G.J., Jordanova E.S. (2012). HLA-E expression in cervical adenocarcinomas: Association with improved long-term survival. J. Transl. Med..

[217] Das D., Sarkar B., Mukhopadhyay S., Banerjee C., Mondal S.B. (2018). An Altered Ratio of CD4+ And CD8+ T Lymphocytes in Cervical Cancer Tissues and Peripheral Blood—A Prognostic Clue?. Asian Pac. J. Cancer Prev..

[218] Sheu B.-C., Hsu S.-M., Ho H.-N., Lin R.-H., Torng P.-L., Huang S.-C. (1999). Reversed CD4/CD8 ratios of tumor-infiltrating lymphocytes are correlated with the progression of human cervical carcinoma. Cancer.

[219] Jiang W., Kang L., Lu H.-Z., Pan X., Lin Q., Pan Q., Xue Y., Weng X., Tang Y.-W. (2004). Normal Values for CD4 and CD8 Lymphocyte Subsets in Healthy Chinese Adults from Shanghai. Clin. Vaccine Immunol..

[220] Shah W., Yan X., Jing L., Zhou Y., Chen H., Wang Y. (2011). A reversed CD4/CD8 ratio of tumor-infiltrating lymphocytes and a high percentage of CD4+FOXP3+ regulatory T cells are significantly associated with clinical outcome in squamous cell carcinoma of the cervix. Cell. Mol. Immunol..

[221] Zhang J., Meng S., Zhang X., Shao K., Lin C. (2022). Infiltration Patterns of Cervical Epithelial Microenvironment Cells During Carcinogenesis. Front. Immunol..

[222] Lin W., Niu Z., Zhang H., Kong Y., Wang Z., Yang X., Yuan F. (2019). Imbalance of Th1/Th2 and Th17/Treg during the development of uterine cervical cancer. Int. J. Clin. Exp. Pathol..

[223] Nishimura T., Nakui M., Sato M., Iwakabe K., Kitamura H., Sekimoto M., Ohta A., Koda T., Nishimura S. (2000). The critical role of Th1-dominant immunity in tumor immunology. Cancer Chemother. Pharmacol..

[224] van der Burg S.H., Piersma S.J., de Jong A., van der Hulst J.M., Kwappenberg K.M.C., van den Hende M., Welters M.J.P., Van Rood J.J., Fleuren G.J., Melief C.J.M. (2007). Association of cervical cancer with the presence of CD4+ regulatory T cells specific for human papillomavirus antigens. Proc. Natl. Acad. Sci. USA.

[225] Maskey N., Thapa N., Maharjan M., Shrestha G., Maharjan N., Cai H., Liu S. (2019). Infiltrating CD4 and CD8 lymphocytes in HPV infected uterine cervical milieu. Cancer Manag. Res..

[226] Sanif R., Nurwany R. (2019). Prognostic significance of CD4/CD8 ratio in patients with advanced cervical cancer. J. Phys. Conf. Ser..

[227] Zhang Y., Yu M., Jing Y., Cheng J., Zhang C., Cheng L., Lu H., Cai M.-C., Wu J., Wang W. (2021). Baseline immunity and impact of chemotherapy on immune microenvironment in cervical cancer. Br. J. Cancer.

[228] Li L., Xu X.T., Wang L.L., Qin S.B., Zhou J.Y. (2021). Expression and clinicopathological significance of Foxp3 and VISTA in cervical cancer. Am. J. Transl. Res..

[229] Wu M.-Y., Kuo T.-Y., Ho H.-N. (2011). Tumor-infiltrating lymphocytes contain a higher proportion of FOXP3+ T lymphocytes in cervical cancer. J. Formos. Med. Assoc..

[230] Ni H., Zhang H., Li L., Huang H., Guo H., Zhang L., Li C., Xu J.-X., Nie C.-P., Li K. (2022). T cell-intrinsic STING signaling promotes regulatory T cell induction and immunosuppression by upregulating FOXP3 transcription in cervical cancer. J. ImmunoTherapy Cancer.

[231] Nedergaard B.S., Ladekarl M., Thomsen H.F., Nyengaard J.R., Nielsen K. (2007). Low density of CD3+, CD4+ and CD8+ cells is associated with increased risk of relapse in squamous cell cervical cancer. Br. J. Cancer.

[232] Hong H.S., Mbah N.E., Shan M., Loesel K., Lin L., Sajjakulnukit P., Correa L.O., Andren A., Lin J., Hayashi A. (2022). OXPHOS promotes apoptotic resistance and cellular persistence in TH17 cells in the periphery and tumor microenvironment. Sci. Immunol..

[233] Alves J.J.P., De Medeiros Fernandes T.A.A., De Araújo J.M.G., Cobucci R.N.O., Lanza D.C.F., Bezerra F.L., Andrade V.S., Fernandes J.V. (2018). Th17 response in patients with cervical cancer. Oncol. Lett..

[234] Oomizu S., Arikawa T., Niki T., Kadowaki T., Ueno M., Nishi N., Yamauchi A., Hirashima M. (2012). Galectin-9 suppresses Th17 cell development in an IL-2-dependent but Tim-3-independent manner. Clin. Immunol..

[235] Punt S., van Vliet M.E., Spaans V.M., de Kroon C.D., Fleuren G.J., Gorter A., Jordanova E.S. (2015). FoxP3(+) and IL-17(+) cells are correlated with improved prognosis in cervical adenocarcinoma. Cancer Immunol. Immunother..

[236] Yu Q., Lou X.-M., He Y. (2015). Preferential Recruitment of Th17 Cells to Cervical Cancer via CCR6-CCL20 Pathway. PLoS ONE.

[237] Mauri C., Bosma A. (2012). Immune Regulatory Function of B Cells. Annu. Rev. Immunol..

[238] Inoue S., Leitner W.W., Golding B., Scott D. (2006). Inhibitory Effects of B Cells on Antitumor Immunity. Cancer Res..

[239] Sarvaria A., Madrigal J.A., Saudemont A. (2017). B cell regulation in cancer and anti-tumor immunity. Cell. Mol. Immunol..

[240] Kim S.S., Shen S., Miyauchi S., Sanders P.D., Franiak-Pietryga I., Mell L., Gutkind J.S., Cohen E.E.W., Califano J.A., Sharabi A.B. (2020). B Cells Improve Overall Survival in HPV-Associated Squamous Cell Carcinomas and Are Activated by Radiation and PD-1 Blockade. Clin. Cancer Res..

[241] Chen Z., Zhu Y., Du R., Pang N., Zhang F., Dong D., Ding J., Ding Y. (2019). Role of Regulatory B Cells in the Progression of Cervical Cancer. Mediat. Inflamm..

[242] Vora C., Gupta S. (2018). Targeted therapy in cervical cancer. ESMO Open..

[243] Harper D.M., Vierthaler S.L., Santee J.A. (2010). Review of Gardasil. J. Vaccines Vaccin..

[244] Monie A., Hung C.F., Roden R., Wu T.C. (2008). Cervarix: A vaccine for the prevention of HPV 16, 18-associated cervical cancer. Biologics.

[245] Grau-Bejar J.F., Garcia-Duran C., Garcia-Illescas D., Mirallas O., Oaknin A. (2023). Advances in immunotherapy for cervical cancer. Ther. Adv. Med. Oncol..

[246] Shao D.-D., Meng F.-Z., Liu Y., Xu X.-Q., Wang X., Hu W.-H., Hou W., Ho W.-Z. (2021). Poly(dA:dT) Suppresses HSV-2 Infection of Human Cervical Epithelial Cells Through RIG-I Activation. Front. Immunol..

[247] Rehwinkel J., Gack M.U. (2020). RIG-I-like receptors: Their regulation and roles in RNA sensing. Nat. Rev. Immunol..

[248] Shi F., Su J., Wang J., Liu Z., Wang T. (2021). Activation of STING inhibits cervical cancer tumor growth through enhancing the anti-tumor immune response. Mol. Cell. Biochem..

[249] Li L., Kim S., Herndon J.M., Goedegebuure P., Belt B.A., Satpathy A.T., Fleming T.P., Hansen T.H., Murphy K.M., Gillanders W.E. (2012). Cross-dressed CD8α+/CD103+ dendritic cells prime CD8+ T cells following vaccination. Proc. Natl. Acad. Sci. USA.

[250] Williford J.-M., Ishihara J., Ishihara A., Mansurov A., Hosseinchi P., Marchell T.M., Potin L., Swartz M.A., Hubbell J.A. (2019). Recruitment of CD103+ dendritic cells via tumor-targeted chemokine delivery enhances efficacy of checkpoint inhibitor immunotherapy. Sci. Adv..

[251] Salmon H., Idoyaga J., Rahman A., Leboeuf M., Remark R., Jordan S., Casanova-Acebes M., Khudoynazarova M., Agudo J., Tung N. (2016). Expansion and Activation of CD103(+) Dendritic Cell Progenitors at the Tumor Site Enhances Tumor Responses to Therapeutic PD-L1 and BRAF Inhibition. Immunity.

[252] Langers I., Renoux V., Reschner A., Touze A., Coursaget P., Boniver J., Koch J., Delvenne P., Jacobs N. (2014). Natural killer and dendritic cells collaborate in the immune response induced by the vaccine against uterine cervical cancer. Eur. J. Immunol..

[253] Soca Gallego L., Dominguez A., Parmar M. (2023). Human Papilloma Virus Vaccine. StatPearls.

[254] Chan J.K., Deng W., Higgins R.V., Tewari K.S., Bonebrake A.J., Hicks M., Gaillard S., Ramirez P.T., Chafe W., Monk B.J. (2017). A phase II evaluation of brivanib in the treatment of persistent or recurrent carcinoma of the cervix: An NRG Oncology/Gynecologic Oncology Group study. Gynecol. Oncol..

[255] Santin A.D., Deng W., Frumovitz M., Buza N., Bellone S., Huh W., Khleif S., Lankes H.A., Ratner E.S., O’Cearbhaill R.E. (2020). Phase II evaluation of nivolumab in the treatment of persistent or recurrent cervical cancer (NCT02257528/NRG-GY002). Gynecol. Oncol..

[256] Naumann R.W., Oaknin A., Meyer T., Lopez-Picazo J.M., Lao C., Bang Y.J., Boni V., Sharfman W.H., Park J.C., Devriese L.A. (2019). Efficacy and safety of nivolumab (Nivo) + ipilimumab (Ipi) in patients (pts) with recurrent/metastatic (R/M) cervical cancer: Results from CheckMate 358. Ann. Oncol..

[257] Liu K., Zhu Y., Zhou Y., Zhu H. (2023). Cemiplimab as Second-Line Therapy for Patients with Recurrent Cervical Cancer: A United States-based Cost-effectiveness Analysis. Adv. Ther..

[258] Zheng X., Gu H., Cao X., Pan B., Xiang H., Ju M., Xu S., Zheng M. (2023). Tislelizumab for cervical cancer: A retrospective study and analysis of correlative blood biomarkers. Front. Immunol..

[259] Basu P., Mehta A., Jain M., Gupta S., Nagarkar R.V., John S., Petit R. (2018). A Randomized Phase 2 Study of ADXS11-001 Listeria monocytogenes-Listeriolysin O Immunotherapy with or without Cisplatin in Treatment of Advanced Cervical Cancer. Int. J. Gynecol. Cancer.

[260] Galicia-Carmona T., Arango-Bravo E., Serrano-Olvera J.A., Flores-de La Torre C., Cruz-Esquivel I., Villalobos-Valencia R., Morán-Mendoza A., Castro-Eguiluz D., Cetina-Pérez L. (2021). ADXS11-001 LM-LLO as specific immunotherapy in cervical cancer. Hum. Vaccin. Immunother..

[261] Hong D.S., Concin N., Vergote I., de Bono J.S., Slomovitz B.M., Drew Y., Arkenau H.T., Machiels J.P., Spicer J.F., Jones R. (2020). Tisotumab Vedotin in Previously Treated Recurrent or Metastatic Cervical Cancer. Clin. Cancer Res..

[262] Song X., Li R., Wang H., Song P., Guo W., Chen Z.S. (2022). Tisotumab vedotin for the treatment of cervical carcinoma. Drugs Today.

[263] Sherer M.V., Kotha N.V., Williamson C., Mayadev J. (2022). Advances in immunotherapy for cervical cancer: Recent developments and future directions. Int. J. Gynecol. Cancer.

[264] Colombo N., Dubot C., Lorusso D., Caceres M.V., Hasegawa K., Shapira-Frommer R., Tewari K.S., Salman P., Hoyos Usta E., Yañez E. (2021). Pembrolizumab for Persistent, Recurrent, or Metastatic Cervical Cancer. N. Engl. J. Med..

[265] Youn J.W., Hur S.Y., Woo J.W., Kim Y.M., Lim M.C., Park S.Y., Seo S.S., No J.H., Kim B.G., Lee J.K. (2020). Pembrolizumab plus GX-188E therapeutic DNA vaccine in patients with HPV-16-positive or HPV-18-positive advanced cervical cancer: Interim results of a single-arm, phase 2 trial. Lancet Oncol..

[266] Zhang L., Wang K., Huang Y., Zhang H., Zhou L., Li A., Sun Y. (2022). Photosensitizer-induced HPV16 E7 nanovaccines for cervical cancer immunotherapy. Biomaterials.

[267] van Meir H., Nout R.A., Welters M.J.P., Loof N.M., de Kam M.L., van Ham J.J., Samuels S., Kenter G.G., Cohen A.F., Melief C.J.M. (2017). Impact of (chemo)radiotherapy on immune cell composition and function in cervical cancer patients. OncoImmunology.

[268] June C.H., O’Connor R.S., Kawalekar O.U., Ghassemi S., Milone M.C. (2018). CAR T cell immunotherapy for human cancer. Science.

[269] Bonini C., Mondino A. (2015). Adoptive T-cell therapy for cancer: The era of engineered T cells. Eur. J. Immunol..

[270] Ribas A., Wolchok J.D. (2018). Cancer immunotherapy using checkpoint blockade. Science.

[271] Ellis G.I., Sheppard N.C., Riley J.L. (2021). Genetic engineering of T cells for immunotherapy. Nat. Rev. Genet..

[272] Etxeberria I., Olivera I., Bolaños E., Cirella A., Teijeira Á., Berraondo P., Melero I. (2020). Engineering bionic T cells: Signal 1, signal 2, signal 3, reprogramming and the removal of inhibitory mechanisms. Cell. Mol. Immunol..

[273] Jazaeri A.A., Zsiros E., Amaria R.N., Artz A.S., Edwards R.P., Wenham R.M., Slomovitz B.M., Walther A., Thomas S.S., Chesney J.A. (2019). Safety and efficacy of adoptive cell transfer using autologous tumor infiltrating lymphocytes (LN-145) for treatment of recurrent, metastatic, or persistent cervical carcinoma. J. Clin. Oncol..

[274] Radvanyi L.G. (2015). Tumor-Infiltrating Lymphocyte Therapy: Addressing Prevailing Questions. Cancer J..

[275] Kazemi M.H., Sadri M., Najafi A., Rahimi A., Baghernejadan Z., Khorramdelazad H., Falak R. (2022). Tumor-infiltrating lymphocytes for treatment of solid tumors: It takes two to tango?. Front. Immunol..

[276] Li B. (2022). Why do tumor-infiltrating lymphocytes have variable efficacy in the treatment of solid tumors?. Front. Immunol..

[277] Zheng J., Huang J., Ma W., Yang W., Hu B.A.-O. (2021). The Antitumor Activity of CAR-T-PD1 Cells Enhanced by HPV16mE7-Pulsed and SOCS1-Silenced DCs in Cervical Cancer Models. Cancer Manag. Res..

[278] He Y., Li X.-M., Yin C.-H., Wu Y.-M. (2020). Killing cervical cancer cells by specific chimeric antigen receptor-modified T cells. J. Reprod. Immunol..

[279] Zhang Y., Li X., Zhang J., Mao L. (2020). Novel cellular immunotherapy using NKG2D CAR-T for the treatment of cervical cancer. Biomed. Pharmacother..

[280] Tsimberidou A.-M., Van Morris K., Vo H.H., Eck S., Lin Y.-F., Rivas J.M., Andersson B.S. (2021). T-cell receptor-based therapy: An innovative therapeutic approach for solid tumors. J. Hematol. Oncol..

[281] Li D., Li X., Zhou W.-L., Huang Y., Liang X., Jiang L., Yang X., Sun J., Li Z., Han W.-D. (2019). Genetically engineered T cells for cancer immunotherapy. Signal. Transduct. Target. Ther..

[282] Ghanaat M., Goradel N.H., Arashkia A., Ebrahimi N., Ghorghanlu S., Malekshahi Z.V., Fattahi E., Negahdari B., Kaboosi H. (2021). Virus against virus: Strategies for using adenovirus vectors in the treatment of HPV-induced cervical cancer. Acta Pharmacol. Sin..

[283] Petry K.U. (2014). HPV and cervical cancer. Scand. J. Clin. Lab. Investig..

[284] Castellsagué X. (2008). Natural history and epidemiology of HPV infection and cervical cancer. Gynecol. Oncol..

[285] Bhattacharjee R., Das S.S., Biswal S.S., Nath A., Das D., Basu A., Malik S., Kumar L., Kar S., Singh S.K. (2022). Mechanistic role of HPV-associated early proteins in cervical cancer: Molecular pathways and targeted therapeutic strategies. Crit. Rev. Oncol./Hematol..

[286] Yu S., Li X., Zhang J., Wu S. (2021). Development of a Novel Immune Infiltration-Based Gene Signature to Predict Prognosis and Immunotherapy Response of Patients With Cervical Cancer. Front. Immunol..

[287] Li F., Li C., Cai X., Xie Z., Zhou L., Cheng B., Zhong R., Xiong S., Li J., Chen Z. (2021). The association between CD8+ tumor-infiltrating lymphocytes and the clinical outcome of cancer immunotherapy: A systematic review and meta-analysis. eClinicalMedicine.

[288] Sugimoto C., Murakami Y., Ishii E., Fujita H., Wakao H. (2022). Reprogramming and redifferentiation of mucosal-associated invariant T cells reveal tumor inhibitory activity. eLife.

[289] Stoitzner P., Sparber F., Tripp C.H. (2010). Langerhans cells as targets for immunotherapy against skin cancer. Immunol. Cell Biol..

[290] Bellone S., El-Sahwi K., Cocco E., Casagrande F., Cargnelutti M., Palmieri M., Bignotti E., Romani C., Silasi D.A., Azodi M. (2009). Human papillomavirus type 16 (HPV-16) virus-like particle L1-specific CD8+ cytotoxic T lymphocytes (CTLs) are equally effective as E7-specific CD8+ CTLs in killing autologous HPV-16-positive tumor cells in cervical cancer patients: Implications for L1 dendritic cell-based therapeutic vaccines. J. Virol..

[291] Lv Y., Ma X., Ma Y., Du Y., Feng J. (2022). A new emerging target in cancer immunotherapy: Galectin-9 (LGALS9). Genes. Dis..

[292] Wang L., Zhao Y., Wang Y., Wu X. (2018). The Role of Galectins in Cervical Cancer Biology and Progression. BioMed. Res. Int..

[293] Kandel S., Adhikary P., Li G., Cheng K. (2021). The TIM3/Gal9 signaling pathway: An emerging target for cancer immunotherapy. Cancer Lett..

[294] Kaesler S., Wölbing F., Kempf W.E., Skabytska Y., Köberle M., Volz T., Sinnberg T., Amaral T., Möckel S., Yazdi A. (2019). Targeting tumor-resident mast cells for effective anti-melanoma immune responses. JCI Insight.

[295] Oldford S.A., Marshall J.S. (2015). Mast cells as targets for immunotherapy of solid tumors. Mol. Immunol..

